# Household heating fuels impact on Acute Respiratory Infection (ARI) symptoms among children in Punjab, Pakistan

**DOI:** 10.1186/s12889-023-17044-1

**Published:** 2023-11-30

**Authors:** Amamah Noor, Ammar Aftab, Memuna Aslam, Sara Imanpour

**Affiliations:** 1 School of Humanities and Social Sciences, Department of Economics, Information Technology, University of the Punjab, Lahore, Pakistan; 2 Department of Health Administration, Pennsylvania State University, Harrisburg, PA United States of America

**Keywords:** Acute respiratory infection (ARI), Heating fuels, Indoor air pollutants, Household air pollution (HAP)

## Abstract

Exposure to household air pollution (HAP) accounted for the loss of 86 million healthy lives in 2019, with almost half of all deaths due to lower respiratory infection among children under 5 years of age. Similarly, the situation in Punjab – Pakistan’s largest province – is also not promising. This study was conducted to examine household energy consumption and respiratory symptoms among children under the age of five in rural and urban areas of Punjab. Using data from the Multiple Indicator Cluster Survey (MICS) 2017-18, logistic regression models were applied to the data of a sample of 35,000 children under the age of five living in households with polluting heating fuels. A hypothesis was formulated to investigate the relationship between polluting heating activities and respiratory infections among children under five. Those Children who live in households having traditional space heaters without chimneys are 50% more likely to have symptoms of Acute Respiratory Infection (ARI) compared to those whose households have chimneys with traditional space heaters. When households utilize polluting heating fuel, the likelihood of children experiencing rapid, shortness of breath increases by 49%, and the likelihood of children displaying ARI symptoms characterized by coughing rises by approximately 30%. This study proposed a complete banning of polluting heating activities and replacing it with cleaner ones using financial incentives. It is pertinent to raise awareness campaigns majorly focusing on the guidelines to adopt better heat output with less harmful emissions.

**Supplementary Information:**

The online version contains supplementary material available at 10.1186/s12889-023-17044-1.

## Introduction

In developing countries, Acute Lower Respiratory Infection (ALRI) is a major cause of death in children (under the age of five), and Lower Respiratory Infection (LRI) – a type of pneumonia – is a major cause of death among children of all age groups (neonates aged 0–27 days, post-neonates aged 28–364 days and children aged 1–4 years) [1]. It is noteworthy that around 2.7 billion people worldwide still are using polluting fuels that cause polluting emissions into the environment [2]. These fuels include wood, crops residue, charcoal, animal dung, and coal which are used as an energy source in their cooking and heating activities, and kerosene for lighting, which ultimately creates a smoky environment. This is associated with millions of deaths yearly through Acute Respiratory Illness (ARI) and cancer in children and women [2]. The primary indoor air pollutants from combustion emit health-damaging pollutants, which are mainly carbon monoxide (CO), fine particulate matter (PM2·5), and hydrocarbons [3].

World Health Organization (WHO) defines solid biomass fuels such as wood, animal dung, crop wastes, charcoal, and solid fuels burned as fuels, along with coal and biomass fuels. Among continents, Asia and Africa are among the largest coal consumers, close to 80%, while most of the wood fuels are used in cooking and heating in these developing countries [4]. However, in some regions, especially in Asia, heating is another important activity after cooking, where the majority of rural households burn biomass fuels in open fireplaces or in non-airtight stoves, which results in a high level of indoor air pollution in the presence of poor ventilation system [5].

Like other developing countries, children and women are major victims of Household Air pollution (HAP), particularly in rural areas of Pakistan, which has serious health effects on them. As per Pakistan Demographic Health Survey (PDHS) 2017–2018, the use of clean fuel, which includes electricity and liquefied petroleum gas/natural gas/biogas, are more common in urban areas (88%) than in rural areas (27%). Furthermore, the Pakistan Social and Living Standards Measurement Survey (PSLM) 2018–2019 also shows; overall, 35% of Pakistani households have access to electricity, Liquid Petroleum Gas (LPG), and gas for heating, whereas a significant regional gap has been observed in the different provinces.

Punjab, one of the country’s largest provinces, still has a low percentage, with only 36% of households relying on clean fuels in the majority of its rural areas. On the other hand, the Multiple Indicator Cluster Survey (MICS) 2017–2018 in Punjab depicts the dependence of 60% of the population on clean fuels and technologies for space heating while 39·4% of the population relies on polluting fuels, including wood, animal, and plant residue for heating. The survey also reveals that 3 out of 4 children under five years of age in Punjab suffer from respiratory illness in the two weeks preceding the survey (Bureau of Statistics Punjab 2017-18).

**Fig. 1:**
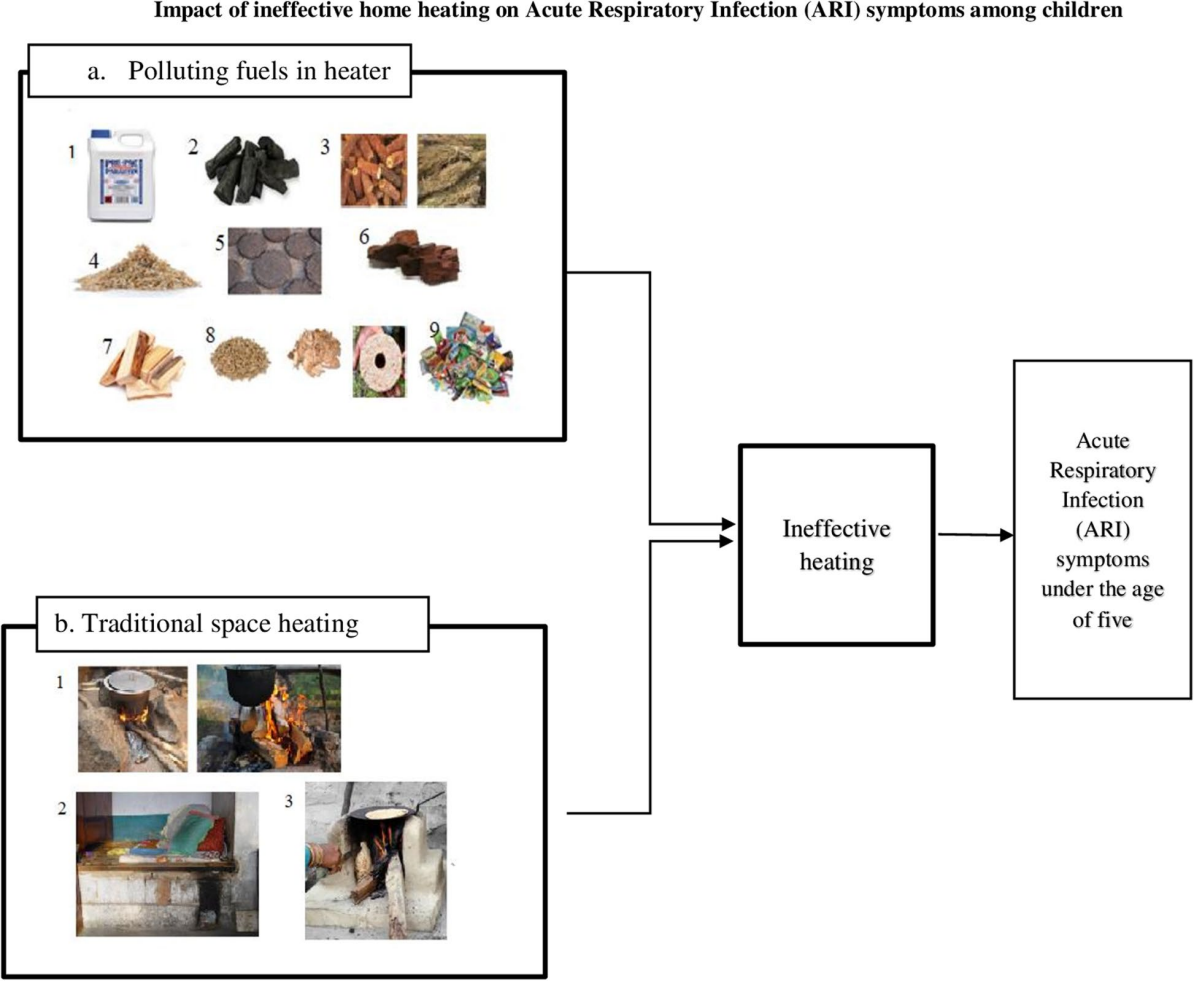
Impact of ineffective home heating on Acute Respiratory Infection (ARI) symptoms among children. 1.Kerosene, 2. Charcoal, 3. Corn cobs & rice straw,4. Sawdust, 5. Animal dung, 6. Coal, 7. Log of firewood, 8. pellets, woodchips, briquettes, 9. Garbage (9). 1. Three-stone stove & Open fire, 2 Traditional heaters, 3. Traditional cook-stove (9)

 Numerous studies have documented climate change and energy security, but only limited academic research has investigated household heating activities and their impact on health, particularly in Pakistan. One of the studies in four districts of Punjab province shows that the use of clean energy sources are limited to basic appliances, and 90% of the respondents used biomass energy sources like crop waste, animal residues, and firewood for cooking and heating [6]. Another study reported that children with polluting fuels in the house are 1.5 times more prone to ARI as compared to children with cleaner fuels in the houses in Pakistan [7]. Farah et al. [8] conducted a study in a rural area of Punjab and observed that the burning of biomass in an open fire causes problems with breathing, coughing, and chest pain in women. Further research is needed to examine the household energy consumption on child health. The cleanliness and pollution level of heating activities in household are determined by the type of fuels used in combination with the technology [9]. This study considers the impact of heating fuels on ARI symptoms among children under the age of five in rural and urban areas of Punjab, Pakistan. The conceptual framework is depicted in Fig. 1.

### Study population and design

**Fig. 2:**
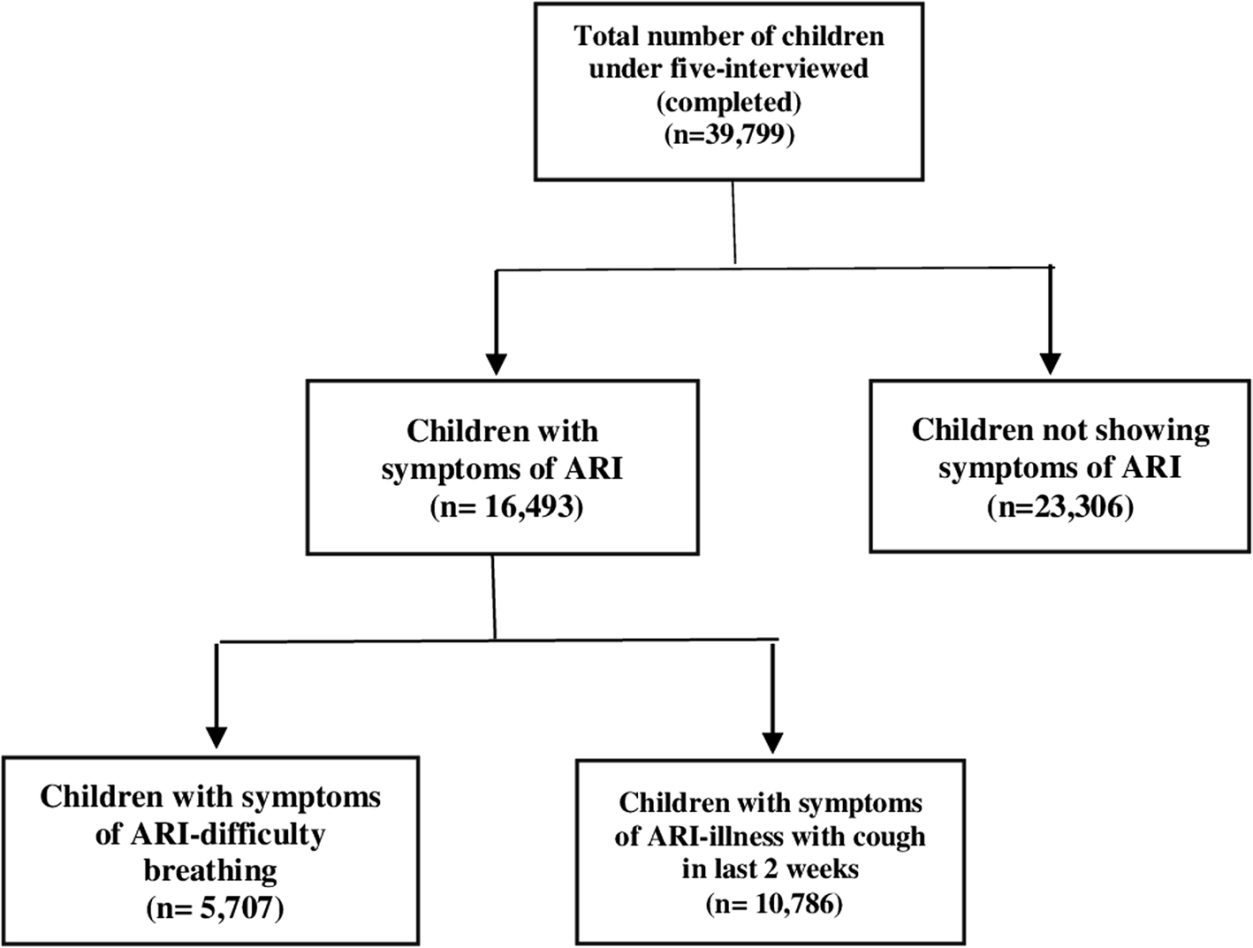
Shows descriptive analysis of the data used in this study by MICS 2017-18

 For this study, Multiple Indicator Cluster Survey (MICS) 2017–2018 was used. It is one of the largest and nationally representative cross-sectional household survey conducted in Pakistan with a sample size of 53,840 households (2692 clusters) and a response rate of 97·9%. A total of 35,000 children under the age of 5 were surveyed. The study sample comprises of children under the age of five, reporting symptoms of ARI in the two weeks preceding the survey. These children belong to the rural and urban areas of Punjab, Pakistan [10]. Figure 2 depicts the sampling details of children under the age of 5 included in the study.

MICS Punjab 2017–2018 uses a two-stage stratified random sampling technique and the sampling frame of Population Census, 2017 was used. For Punjab, the sampling frame consists of 36 administrative districts and within each region, rural and urban areas were identified as the main sampling strata. In the first phase, a specific number of census enumeration areas were selected whereas, in the second phase, a household-level sampling was carried out. Then, using systematic sampling procedure, 25 households were drawn in each sample enumeration area. In the selection of both the enumeration areas and households, a random systematic sampling procedure was used. Additionally, in these households, all women from 15 to 49 years, all men aged 15 to 49 and children under the age of 5 were selected. Additionally, the survey offered key health indicators on both national and regional levels, further encompassing rural and urban areas as well. Therefore, this study restricts itself to one of the health indicators i.e. prevalence of ARI symptoms in children less than 5 years of age as our main dependent variable of interest and household energy consumption as the independent variable from households in rural and urban areas of Punjab, Pakistan [10].

### Variables

Table 1 provides a detailed description of study variables. The definition of ARI used in the Multiple Indicator Cluster Survey (MICS) is based on the mother’s perception either child has a cough, is breathing faster than usual with short, quick breaths or is having difficulty breathing excluding children that had only a blocked nose [11]. Two variables were constructed for ARI i.e., illness with cough and short/rapid/difficulty breathing problem [7, 11, 12]. The main explanatory variables include: *(a) type of fuel and energy source used in heater (b) combined energy source used during heating activity*. Detailed description of variables used in this study is given in Table 1.

**Table 1:** Description of variables used in the study

Variable	Description/Definition- MICS 2017–2018	Variable construction
**Illness with cough**	At any time in the last two weeks preceding the survey, has child had an illness with a cough (yes; no)	**1**: Child suffering from illness with cough
		**0**: Child not suffering from illness with cough
**Rapid/short/difficult breathing**	At any time in the last two weeks preceding the survey, has child had fast, short, rapid breaths or difficulty breathing (yes; no)	**1**: Child suffering from rapid/short/difficult breathing
		**0**: Child not suffering from rapid/short/difficult breathing
**Type of fuel and energy source used in heater**	Respondents living in households with primary reliance on clean fuels and technologies for space heating	**1**: *Polluting energy source* (alcohol/ethanol, gasoline/diesel, kerosene/paraffin, coal/lignite, charcoal, wood, crop residue/grass/straw/shrubs, animal-dung/waste, processed biomass (pellets) or woodchips, garbage/plastic and sawdust)
		**0**:*Non-polluting energy source* (solar air heater, electricity, piped natural gas, liquefied petroleum gas/cooking gas, biogas)
**Type of space heating**	Respondents living in households mainly use space heating when needed	**1**:*Traditional space heater* (space heater, traditional cook-stove and three stone stove/open fire)
		**0**:*Manufactured space heater* (manufactured space heater and manufactured cook-stove)
**Presence of chimney**	Respondents living in households with chimney (yes; no)	**1**:Presence of no chimney in the house
		**0**:Presence of chimney
**Mother’s education**	Women aged 15–49 years by highest level of school attended	**1**:No education attained
		**2**:Primary/Middle education level
		**3**:Secondary/Higher education level
**Mother’s age at birth**	Age of the mother at the time of child’s birth	**1**: <20 years
		**2**: 20–34 years
		**3**: 35 + years
**Child’s Age**	Age of the child (under 5 years)	0,1,2,3,4
**Sex of child**	Gender of the child	**1**: Boy
		**0**: Girl
**Residence**	Region of residence of the child	**1**: Rural
		**0**: Urban

### Models

Logistic regression model was used for this study due to dependent variable being dummy variable. Four models were developed for this study in which dependent variable comprised health outcome as ARI Symptoms. The independent variables in model are *(a) type of fuel and energy source used in heater (b) combined energy source used during heating* in both rural and urban areas of Punjab, Pakistan. In Models 1 and 2, the dependent variables are ARI symptoms (cough with short/rapid/difficulty breathing and cough-related illness), and the independent variables in these models include the type of fuel and energy source used in the heater for heating the house, followed by control variables. For Models 3 and 4, ARI symptoms (cough with short/rapid/difficulty breathing, and illness with cough) were included as a dependent variable whilst using another independent variable i.e. *combined energy source used during heating*, which includes a total of four combinations: type of energy source or type of fuel used in heater, type of space heater used during heating and no of chimney on space heater, followed by control variables. To run analysis for these models, STATA 16 software package was used. Following are the equations for he models:

$$\text{l}\text{o}\text{g}\left({Y}_{ij }\right)$$ = $${\beta }_{0}$$ + $${\beta }_{1}TypeofFuel\&EnergySource$$ + Ω*𝑖*+ *𝛼𝑗*+ $${\in }_{ij}$$

$$\text{l}\text{o}\text{g}\left({Y}_{ij }\right)$$= $${\beta }_{0}$$ + $${\beta }_{1}CombinedEnergySource$$ + Ω*𝑖*+ *𝛼𝑗*+ $${\in }_{ij}$$

where Y_ij_ represents the symptoms of children ‘i’ in the household ‘j’; Ω_i_ represents the control variables at the individual-level, *𝛼*_j_ denotes household level control variables and *𝜀*_ij_ is the error term.

## Results

### Descriptive statistics

Table 2 presents the descriptive statistics of the mother characteristics of the respondents, child characteristics and the residential area. According to the survey data, approximately 27% of children experienced ARI symptoms (cough with illness in the past 2 weeks). Additionally, 14% of children exhibited symptoms of rapid, difficulty, or quick and short breathing, while only 12% of children displayed both ARI symptoms (cough with illness and rapid/difficulty/quick/short breathing problems). Around 40% of household use polluting fuel as the main source of energy in heating activity and have traditional heaters for space heating in household. It must be noted that approximately 91% of households have no chimney on heaters. Furthermore, it was found that the 53% of the children have an uneducated mother with more than half of the mothers belonging to age group 20–34 years. Lastly, it was observed that the distribution of children across different age groups is nearly equal, and approximately 52% of the children in the dataset are male.

**Fig. 3:**
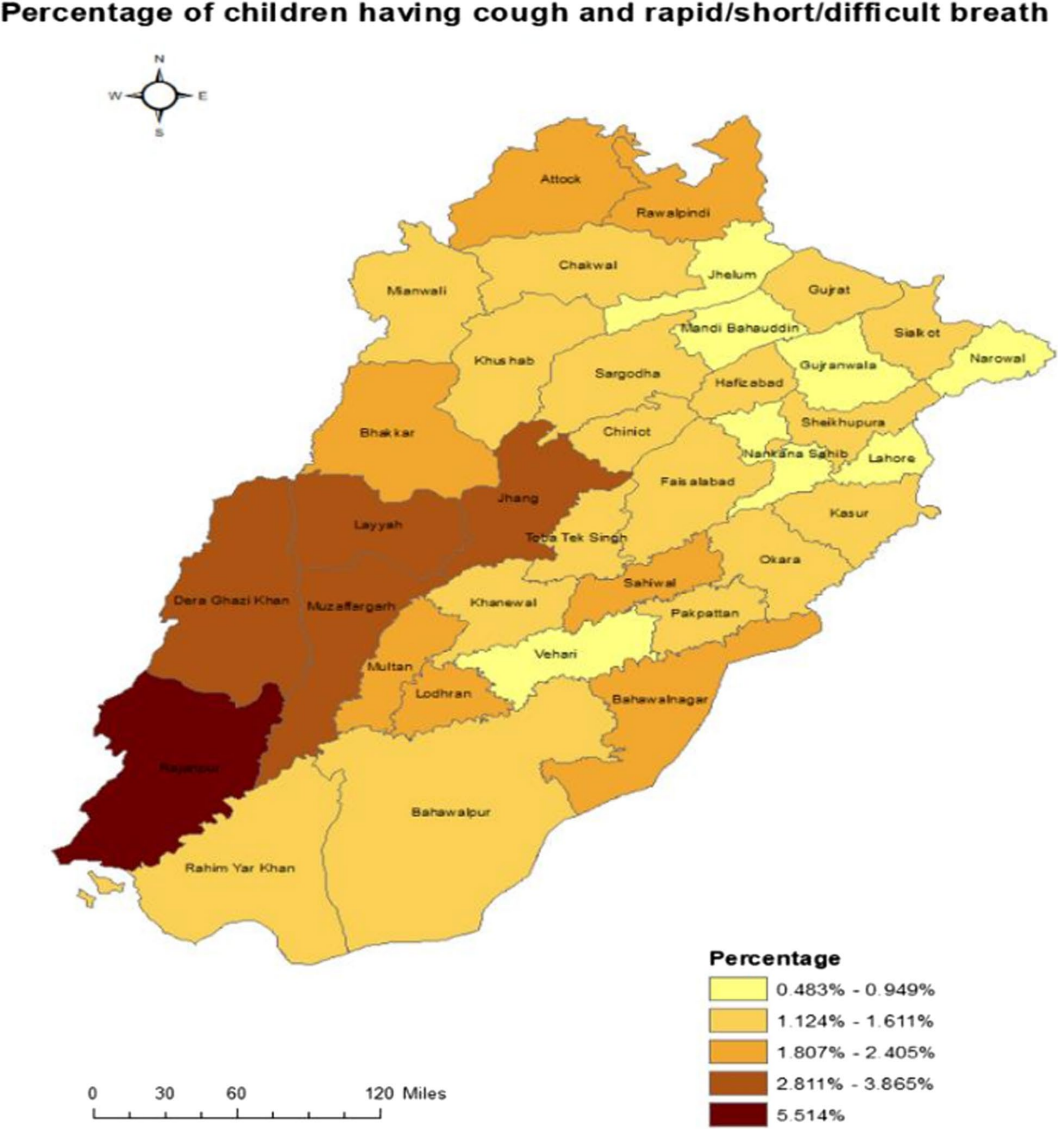
Choropleth map for district wise percentage of children cough and difficult breathing

**Fig. 4:**
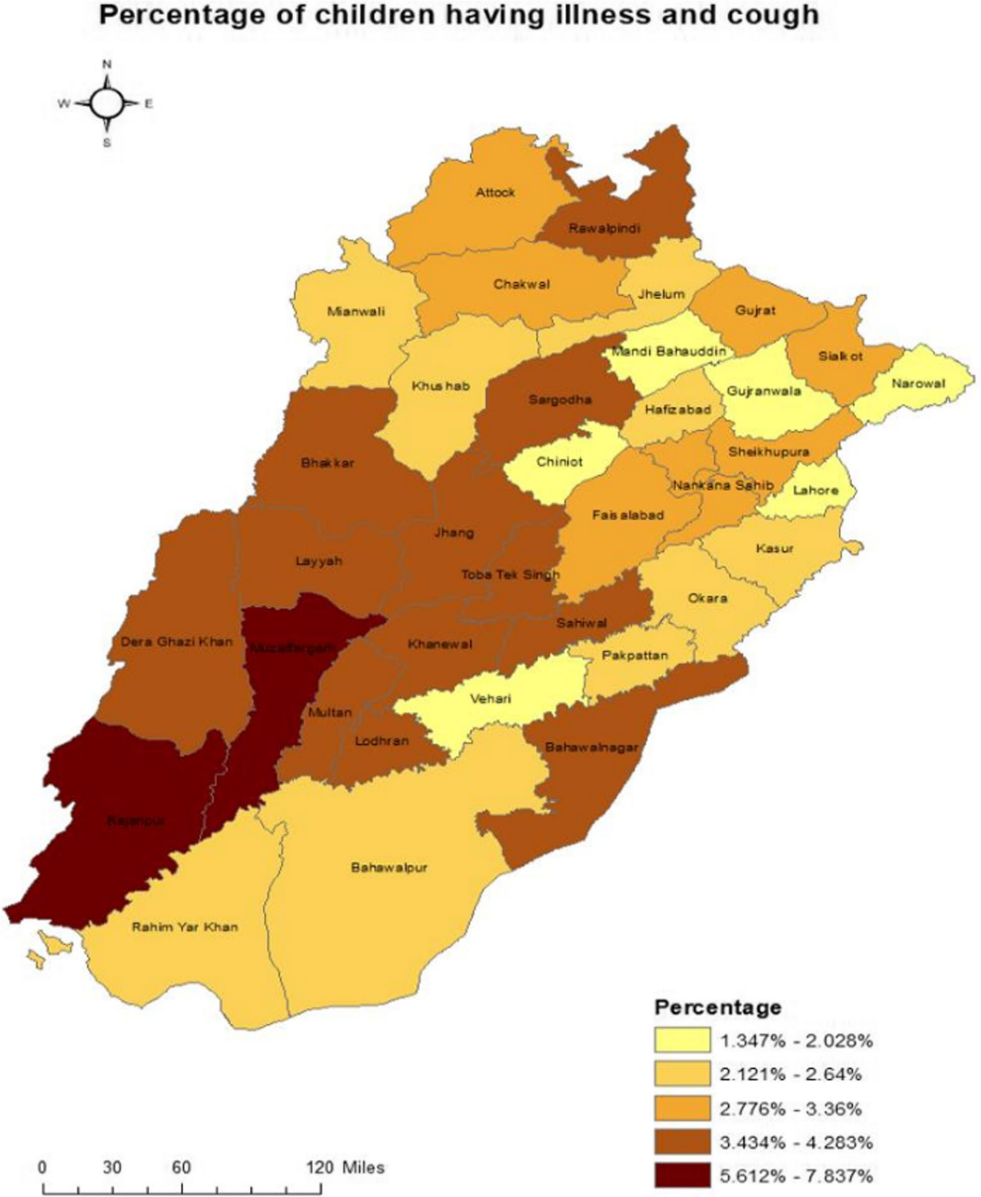
Choropleth map for district wise percentage of children with illness and cough

The following choropleth maps (Figs. 3 and 4) give an overview of the spread of cases of cough with difficulty in breathing across districts. The lighter tone depicts districts with less such cases and darker tone depicts districts with more of such cases.

As presented in the map (Fig. 3), the district with the most cases (of cough with difficulty in breathing) was Rajanpur. Layyah, Muzaffargarh, Dera Ghazi Khan and Jhang also had quite a lot of cases as compared to other districts.

As presented in the map (Fig. 4), the district with the most cases (of cough with illness) is Rajanpur and Muzaffargarh.

**Table 2:** Descriptive statistics

Variable Name	Variable Categories	Percentages (Absolute numbers in parenthesis)
**Symptom of ARI (illness with cough in last 2 weeks)**	No	72·88% (28,991)
	Yes	27·12% (10,786)
**Symptom of ARI (rapid, difficulty or quick and short breathing problem in children)**	No	85.64% (34,039)
	Yes	14·36% (5707)
**Type of energy source for heater**	Non-Polluting Fuel	58·18% (36,432)
	Polluting Fuel	41·82% (26,190)
**Type of space heating in household**	Manufactured Space Heater	58·75% (36,068)
	Traditional Space Heater	41·25% (25,321)
**Space heaters(have a chimney?)**	Presence of Chimney	8·80% (5303)
	No Chimney	91·20% (54,942)
**Mother level of education**	No Education	53·27% (75,195)
	Primary/Middle	27·37% (38,636)
	Secondary/Higher	19·36% (27,321)
**Mother’s age at childbirth**	< 20	11·37% (17,954)
	20–34	80·94% (127,796)
	35+	7·69% (12,149)
**Age of child**	0	20·39% (8125)
	1	19·78% (7881)
	2	19·69% (7847)
	3	20·85% (8310)
	4	19·29% (7686)
**Sex of child**	Girl	48·51% (76,595)
	Boy	51·49% (81,304)
**Area**	Urban	72·08% (255,448)
	Rural	27·92% (98,493)

### Regression analysis

The outcomes of this study are presented in this section with Table 3 encompassing the results using health outcome of ARI symptoms (cough with short/rapid/difficulty breathing, illness with cough and both) as a dependent variable. It must be noted that the table presents the results from all the models.

**Table 3:** Estimating relationship between ARI symptoms and heating sources

Variables	Categories	Model 1	Model 2	Model 3	Model 4
Type of energy source heater	Non-Polluting Fuel	1·00	1·00		
	Polluting Fuel	1·48***	1·30***		
Combined energy source used during heating	Non-Polluting Energy Source in heater, Manufactured space heater and presence of chimney	**Not applicable to Models 1 and 2**		1·00	1·00
	Polluting Energy source in heater, Traditional space heater and no chimney			1·49***	1·29***
	Polluting Energy source in heater, Traditional space heater			1·84***	1·303*
	Polluting Energy source in heater and no chimney			0·903	0·956
	Traditional space heater and no chimney			1·118	1·12
Sex of Child	Girl	1·00	1·00	1·00	1·00
	Boy	1·09***	1·042*	1·078	1·1*
Child Age	0, 1, 2, 3, 4	0·904***	1·042**	0·904***	1·05**
Mother’s Education	No education	1·00	1·00	1·00	1·00
	Primary/Middle	1·03	1·141*	1·038	1·117
	Secondary/Higher	0·77***	0·966	0·769***	0·948
Mother’s Age	Less than 20	1·00	1·00	1·00	1·00
	20 to 34	0·92	0·82*	0·922	0·818*
	35 and more	0·97	0·77**	0·906	0·758**
Residence	Urban	1·00	1·00	1·00	1·00
	Rural	1·04	0·989	0·961	0·952

Dependent variable: ARI symptoms

*** *p* < 0·01, ** *p* < 0·5, * *p* < 0·1

From the statistics, It can be seen that the polluting fuels used in heating activity were statistically significant (*p* < 0·01 and *p* < 0·05). Overall, this study found that children under the age of five living in households with polluting heating fuels, traditional space heater and no chimney on space heater were 1·5 times more likely to have symptoms of short/rapid/difficulty breathing symptoms of ARI whereas children living under these conditions were 1·3 times more likely to have illness with cough symptoms of ARI.

In the Models 1 and 2, the base category is non-polluting heating fuel, and the estimates clearly demonstrated that the odds of children having ARI symptom (rapid/short/difficulty breathing) were higher by 49% when the household use polluting heating fuel, whereas the odds of children with ARI symptom of illness with cough, were higher by around 30% in the presence of polluting heating fuel (*p* < 0·01). Moving forward to Models 3 and 4, it must be added that the base category in this case was the combination of non-polluting energy source or fuel used in heater, manufactured space heater and presence of chimney on heater. In Model 3, the odds of children having the ARI symptoms were higher by 50% (*p* < 0·01) when household use polluting energy source in heater and traditional space heater for heating activity with no chimney on it, while the odds of children having ARI symptoms, showing only illness with cough were higher by 30% (*p* < 0·01). Similarly, around 84% (*p* < 0·01) of children were more likely to have ARI symptoms (short/rapid/difficulty breathing) when the household used polluting heating energy source and, traditional space heater whereas, around 30% of children were more likely to experience the ARI symptom (illness with cough), showing only illness with cough in households with this combination.

It was also found that the gender variable was statistically significant in this study. In particular, the results revealed that the odds of boys having ARI symptoms were higher by 10% (*p* < 0·01) when household use polluting energy source for heating purpose. The models in our study further revealed that with one year increase in age, the odds of having ARI symptom (rapid/short/difficulty breathing) were lower by 10% in the household with the polluting heating fuels; whereas they were around 5% more likely to experience the ARI symptoms (illness with cough) in the presence of polluting heating fuels.

Moreover, mother education in models was also found statistically significant (*p* < 0·01). In all models, the respiratory symptoms of children were found to be less among children with the mothers having education level of secondary/higher level as compared to mothers with primary/middle education level. The study also found that the mother’s age was also statistically significant (*p* < 0·10) only in Models 2 and 4. In these models, the children belonging to the mothers with age group of 20–34 years and 35 + years are less likely to experience to ARI symptoms. Finally, it was noted that the residential factor was statistically insignificant in all the models.

## Discussions

Pakistan is developing country with over 60% of the population living in rural areas. There are varying sources of energy for people living in urban and rural areas across different income groups and type of households (Moeen, et al. 2015). Choice of energy sources can have impact on health of women and children. From the above results, It was comprehended that the polluting fuels used in heating activity were significantly associated with the ARI symptoms among children under the age of five. A high prevalence of ARI symptoms was found among children under the five years of age who used polluting heating fuels as compared with the non-polluting heating fuels users. It must also be added that the result matches with Janjua et al. [13]. which was conducted in Dadu district of Sindh province in Pakistan. Moreover, it was understood that the findings of this study are similar to the previous studies conducted in different countries. In Nepal, children suffering from ARI symptoms were 1.80 times higher among solid fuel users [14]. Similarly, the study conducted in urban slums of Bangladesh showed that respiratory symptoms like cough, chest tightness, and shortness of breath were significantly high in households using biomass fuel in their daily activities [15]. Furthermore, a study conducted in India shows that children from households using polluting fuels for cooking or space heating are at 80% higher risk of ARI symptoms than children from households using cleaner fuels [11].

A number of co-factors were explored in this study which showed a significant association between the ARI symptoms and polluting heating fuels and space heaters under a poor ventilation system. The children under the age of five were more likely to develop ARI symptoms, showing illness with cough when living with the polluting fuels used in heating activity and in the presence of a traditional space heater with no chimney. These results are supported by the published research work in India, Pakistan and Nigeria [11, 13, 16]. However, the findings in terms of gender remain in contradiction with the previous researches conducted in Bangladesh by [17] and in India by [18], where the results depicted an insignificant association for boys with ARI symptoms as compared to girls. A possible explanation might be that girls spend more time with their mother in household rather than boys. In context to maternal education, this study is in line with the previous research conducted in Pakistan by Maheen et al. [19] which identifies that the odds of ARI among children are substantially lower among children of mothers with secondary/higher education as compared to no education. This is possible because children spend more time with their mothers and educated mothers are more aware of their child health quality and take appropriate measures to mitigate the risks the child might be exposed to [20, 21]. Moreover, mother’s age in some of our model was found to be significant. Children of the mother between the age group 20–35 years and 35 + years were found to be at low risk of having ARI symptoms as compared to the mother belonging to the age group of less than 20 years. The results make empirical relevance because younger mothers have weaker immune system, which results in the delivery of children with weaker immune system as well, overall increasing the risk of ARI infections [7]. In Pakistan, a limited number of studies have compared the prevelance of ARI symptoms in rural and urban areas. In this study, results are not significant in the domain of residential factor i.e. rural area. Moreover, many studies in India and Bangladesh show that a higher prevalence of ARI symptoms among children under the age of five are from urban areas as compared to the rural areas, the reason of which is identified as overcrowding in these urban areas or the difference in socio-economic factors and cultural factors present in different geographical regions of a country [22, 23].

We also found out that the ARI sympyoms were higher among children in the Western part of the Punjab region. This is mainly due to the western part of the Punjab boarder that closed to mountains range and the increasing dry seasonal pattern might cause the increased odds of children suffering from ARI [24].

Even though this study, which investigates the impact of household energy consumption during heating activities on children’s ARI symptoms under the age of 5, is first of its kind in Pakistan, but it has some limitations. First, the collection of data on the sources of heating fuel may be a source of miscommunication bias as many households in rural areas use the combination of fuels in their household activities. The MICS only collects information on the primary fuel usage while no information on secondary fuel was collected. Second, this study was not able to control for many other child characteristics such as vaccination status, birth order and child breast-feeding status due to unavailability of data. Finally, it is added that this research is limited to the district wise data only in one province of Pakistan. Therefore, it is quite likely for the research to produce different results for the different provinces depending upon their socio-economic conditions.

## Conclusion

Household air pollution is also the important contributor to the external air pollution. It is proposed to put a ban on polluting/technology and replacing it with manufactured/cleaner heating activities, but many rural households in Pakistan will be unlikely to afford it. Therefore, this policy should focus on financial incentives. It is the need of the hour to run public awareness campaigns similar to Sweden, where 73% of the respondents followed guidelines and shifted from old methods of burning polluting fuel [25]. These awareness campaigns majorly focused on the guidelines to adopt better heat output with less harmful emissions, such as burning the right fuel, in the right amount, using the right techniques [26]. For such interventions and programs to be effective, it is pertinent to note that local needs and community participation is needed through municipal government and several different non-governmental organizations (NGOs).

### Supplementary Information

**Additional file 1.**

## Data Availability

All the relevant data sets used for statistical and descriptive analysis, graphs are openly available on the UNICEF website: https://mics.unicef.org/surveys.
