# Supplementary material for: Household heating fuels impact on Acute Respiratory Infection (ARI) symptoms among children in Punjab, Pakistan
Source: BMC Public Health. 2023 Nov 30;23:2380. doi: 10.1186/s12889-023-17044-1 (PMC10691043; doi:10.1186/s12889-023-17044-1)
Supplement: Supplementary file 1 — Additional file 1. [file 12889_2023_17044_MOESM1_ESM.pdf]

HOUSEHOLD QUESTIONNAIRE

Multiple Indicator Cluster Survey (MICS) Punjab, 2017

HOUSEHOLD INFORMATION PANEL		HH
**HH1**. *Cluster number:* ___ ___	**HH2**. *Household number:* ___ ___	
**HH3**. *Interviewer's name and number:*<br>NAME ____________________ ___ ___	**HH4**. *Supervisor's name and number:*<br>NAME ____________________ ___ ___ ___ ___	
**HH5**. *Day / Month / Year of interview:*<br>___ ___ /___ ___ / 2 0 1 __	**HH7.** *District code:*____________________ ___ ___	
**HH6**. *Area:*	URBAN .... 1<br>RURAL 2	
**HH8**. *Is the household selected for Questionnaire for Men?*	YES .... 1<br>NO 2	
**HH9**. *Is the household selected for Water Quality Testing?*	YES .... 1<br>NO 2	
**HH10**. *Is the household selected for blank testing?*	YES .... 1<br>NO 2	

Check that the respondent is a knowledgeable member of the household and at least 18 years old before proceeding. You may only interview a child age 15-17 if there is no adult member of the household or all adult members are incapacitated. You may not interview a child under age 15.	**HH11**. *Record the time.*<br>HOURS : MINUTES<br>__ __ : __ __

HH12. Assalam O Alaikum, my name is (***your name***). We are from Bureau of Statistics, Planning & Development Department, Government of the Punjab, Lahore. We are conducting a survey about the situation of children, families and households. I would like to talk to you about these subjects. This interview usually takes about 40 minutes. Following this, I may ask to conduct additional interviews with you or other individual members of your household. All the information we obtain will remain strictly confidential and anonymous. If you do not wish to answer a question or stop the interview, please let me know. May I start now?

YES .... 1<br>NO / NOT ASKED 2	1⇨*LIST OF HOUSEHOLD MEMBERS*<br>2⇨*HH46*

**HH46**. *Result of Household Questionnaire interview:*<br><br>*Discuss any result not completed with Supervisor.*	COMPLETED .... 01<br>NO HOUSEHOLD MEMBER AT HOME OR NO COMPETENT RESPONDENT AT HOME AT TIME OF VISIT .... 02<br>ENTIRE HOUSEHOLD ABSENT FOR EXTENDED PERIOD OF TIME .... 03<br>REFUSED .... 04<br>DWELLING VACANT OR ADDRESS NOT A DWELLING .... 05<br>DWELLING DESTROYED .... 06<br>DWELLING NOT FOUND .... 07<br><br>OTHER (*specify*) ____________________ 96

**HH47**. *Name and line number of the respondent to Household Questionnaire interview:*<br><br>NAME____________________ ___ ___	*To be filled after the Household Questionnaire is completed*		*To be filled after <u>all</u> the questionnaires are completed*	
	TOTAL NUMBER		COMPLETED NUMBER	
HOUSEHOLD MEMBERS	**HH48**	__ __		
WOMEN AGE 15-49	**HH49**	__ __	**HH53**	__ __
If household is selected for Questionnaire for Men:<br>MEN AGE 15-49	**HH50**	__ __	**HH54**	__ __
CHILDREN UNDER AGE 5	**HH51**	__ __	**HH55**	__ __
CHILDREN AGE 5-17	**HH52**	__ __	**HH56**	ZERO ...... 0<br>ONE 1

LIST OF HOUSEHOLD MEMBERS	HL

First complete HL2 for all members of the household. Then proceed with HL3 and HL4 vertically. Once HL2-HL4 are complete for all members, make sure to probe for additional members: Those that are not currently at home, any infants or small children and any others who may not be family (such as servants, friends) but who usually live in the household. Then, ask questions HL5-HL20 for each member one at a time. If additional questionnaires are used, indicate by ticking this box:..

HL1. Line No.	**HL2**. First, please tell me the name of each person who usually lives here, starting with the head of the household.<br><br>*Probe for additional household members.*	**HL3**. What is the relation-ship of ***(name)*** to (***name of the head of house hold***)?	**HL4**. What is the sex of ***(name)***?<br><br>1 MALE<br>2 FEMALE<br>3 TRANS-GENDER	**HL5**. What is ***(name)***'s date of birth?<br><br>98 DK	 <br><br>9998 DK	**HL6**. How old is ***(name)***?<br><br>*Record in completed years.*<br><br>*If age is 95 or above, record '95'.*	**HL7**. Did ***(name)*** stay here last night?<br><br>1 YES<br>2 NO	***HL7A***. *Age 10 and above?*<br><br>1 YES<br>2 NO ⇨ *HL10*	**HL7B**. What is marital status of ***(name)***?<br><br>1 Married<br>2 Widowed<br>3 Divorced<br>4 Separated<br>5 Never Married<br>8 DK	***HL8***. *Record line number if woman and age 15-49.*	***HL9***. *Record line number if man, age 15-49 and HH8 is yes.*	***HL10***. *Record line number if age 0-4*	***HL11***. *Age 0-17?*<br><br>1 YES<br>2 NO ⇨ *Next Line*	**HL12**. Is ***(name)***'s natural mother alive?<br><br>1 YES<br>2 NO ⇨ *HL16*<br>8 DK ⇨ *HL16*	**HL13**. Does ***(name)***'s natural mother live in this household?<br><br>1 YES<br>2 NO ⇨ *HL15*	***HL14***. *Record the line number of mother and go to HL16.*	**HL15**. Where does ***(name)***'s natural mother live?<br><br>1 Abroad<br>2 In another household in the same district<br>3 In another household in another district<br>4 Institution in this country<br>8 DK	**HL16**. Is ***(name)***'s natural father alive?<br><br>1 YES<br>2 NO ⇨ *HL20*<br>8 DK ⇨ *HL20*	**HL17**. Does ***(name)***'s natural father live in this household?<br><br>1 YES<br>2 NO ⇨ *HL19*	***HL18***. *Record the line number of father and go to HL20.*	**HL19**. Where does ***(name)***'s natural father live?<br><br>1 Abroad<br>2 In another household in the same district<br>3 In another household in another district<br>4 Institution in this country<br>8 DK	**HL20**. *Copy the line number of mother from HL14. If blank, ask:*<br><br>Who is the primary caretaker of ***(name)***?<br><br>*If 'No one' for a child age 15-17, record '90'.*
Line	Name	Relation*	M F T	month	Year	Age	Y N	Y N	Marital Status	W 15-49	M 15-49	0-4	Y N	Y N DK	Y N	Mother		Y N DK	Y N	Father		
01		<u>0 1</u>	1 2 3	__ __	__ __	__ __	1 2	1 2	1 2 3 4 5 8	01	01	01	1 2	1 2 8	1 2	__ __	1 2 3 4 8	1 2 8	1 2	__ __	1 2 3 4 8	__ __
02		__ __	1 2 3	__ __	__ __	__ __	1 2	1 2	1 2 3 4 5 8	02	02	02	1 2	1 2 8	1 2	__ __	1 2 3 4 8	1 2 8	1 2	__ __	1 2 3 4 8	__ __
03		__ __	1 2 3	__ __	__ __	__ __	1 2	1 2	1 2 3 4 5 8	03	03	03	1 2	1 2 8	1 2	__ __	1 2 3 4 8	1 2 8	1 2	__ __	1 2 3 4 8	__ __
04		__ __	1 2 3	__ __	__ __	__ __	1 2	1 2	1 2 3 4 5 8	04	04	04	1 2	1 2 8	1 2	__ __	1 2 3 4 8	1 2 8	1 2	__ __	1 2 3 4 8	__ __
05		__ __	1 2 3	__ __	__ __	__ __	1 2	1 2	1 2 3 4 5 8	05	05	05	1 2	1 2 8	1 2	__ __	1 2 3 4 8	1 2 8	1 2	__ __	1 2 3 4 8	__ __
06		__ __	1 2 3	__ __	__ __	__ __	1 2	1 2	1 2 3 4 5 8	06	06	06	1 2	1 2 8	1 2	__ __	1 2 3 4 8	1 2 8	1 2	__ __	1 2 3 4 8	__ __
07		__ __	1 2 3	__ __	__ __	__ __	1 2	1 2	1 2 3 4 5 8	07	07	07	1 2	1 2 8	1 2	__ __	1 2 3 4 8	1 2 8	1 2	__ __	1 2 3 4 8	__ __
08		__ __	1 2 3	__ __	__ __	__ __	1 2	1 2	1 2 3 4 5 8	08	08	08	1 2	1 2 8	1 2	__ __	1 2 3 4 8	1 2 8	1 2	__ __	1 2 3 4 8	__ __
09		__ __	1 2 3	__ __	__ __	__ __	1 2	1 2	1 2 3 4 5 8	09	09	09	1 2	1 2 8	1 2	__ __	1 2 3 4 8	1 2 8	1 2	__ __	1 2 3 4 8	__ __
10		__ __	1 2 3	__ __	__ __	__ __	1 2	1 2	1 2 3 4 5 8	10	10	10	1 2	1 2 8	1 2	__ __	1 2 3 4 8	1 2 8	1 2	__ __	1 2 3 4 8	__ __
11		__ __	1 2 3	__ __	__ __	__ __	1 2	1 2	1 2 3 4 5 8	11	11	11	1 2	1 2 8	1 2	__ __	1 2 3 4 8	1 2 8	1 2	__ __	1 2 3 4 8	__ __
12		__ __	1 2 3	__ __	__ __	__ __	1 2	1 2	1 2 3 4 5 8	12	12	12	1 2	1 2 8	1 2	__ __	1 2 3 4 8	1 2 8	1 2	__ __	1 2 3 4 8	__ __
13		__ __	1 2 3	__ __	__ __	__ __	1 2	1 2	1 2 3 4 5 8	13	13	13	1 2	1 2 8	1 2	__ __	1 2 3 4 8	1 2 8	1 2	__ __	1 2 3 4 8	__ __
14		__ __	1 2 3	__ __	__ __	__ __	1 2	1 2	1 2 3 4 5 8	14	14	14	1 2	1 2 8	1 2	__ __	1 2 3 4 8	1 2 8	1 2	__ __	1 2 3 4 8	__ __
15		__ __	1 2 3	__ __	__ __	__ __	1 2	1 2	1 2 3 4 5 8	15	15	15	1 2	1 2 8	1 2	__ __	1 2 3 4 8	1 2 8	1 2	__ __	1 2 3 4 8	__ __

** Codes for **HL3**: Relationship to head of household:*

01 Head	05 Grandchild	09 Brother-in-law / Sister-in-law	13. Adopted /Foster / Stepchild
02 Spouse/ Wife	06 Parent	10 Uncle/Aunt	14. Servant (live in)
03 Son/ Daughter	07 Parent-in-law	11 Niece / Nephew	96. Other (Not related)
04 Son in law/ Daughter in law	08 Brother / sister	12 Other Relative	98. DK

EDUCATION 1															ED
**ED1**.<br>*Line number*	**ED2**.<br>*Name and age.*<br><br>*Copy names and ages of <u>all</u> members of the household from HL2 and HL6 to below <u>and</u> to next page of the module.*		**ED3**.<br>*Age 3 or above?*<br><br>1 YES<br>2 NO ⇨<br>*Next Line*		**ED4**.<br>Has ***(name)*** ever attended school or any PreSchool/ Katchi/ Early Childhood Education programme?<br><br>1 YES<br>2 NO ⇨<br>*Next Line*		**ED5**.<br>What is the highest level and grade or class of school ***(name)*** has ever <u>attended</u>?<br><br>LEVEL:<br>0 PRESCHOOL/KATCHI /ECE ⇨ *ED7*<br>1 PRIMARY<br>2 LOWER SECONDARY<br>3 UPPER SECONDARY<br>4 HIGHER<br>8 DK	GRADE/CLASS:<br>98 DK ⇨ *ED7*	**ED6**.<br>Did ***(name)*** ever <u>complete</u> that (grade/ class)?<br><br>1 YES<br>2 NO<br>8 DK			**ED7**.<br>*Age 3-24?*<br><br>1 YES<br>2 NO ⇨<br>*Next Line*		**ED8**.<br>*Check ED4: Ever attended school or PreSchool/ Katchi /ECE?*<br><br>1 YES<br>2 NO ⇨<br>*Next Line*	
LINE	NAME	AGE	YES	NO	YES	NO	LEVEL	GRADE/ CLASS*	Y	N	DK	YES	NO	YES	NO
01		___ ___	1	2	1	2	0 1 2 3 4 8	___ ___	1	2	8	1	2	1	2
02		___ ___	1	2	1	2	0 1 2 3 4 8	___ ___	1	2	8	1	2	1	2
03		___ ___	1	2	1	2	0 1 2 3 4 8	___ ___	1	2	8	1	2	1	2
04		___ ___	1	2	1	2	0 1 2 3 4 8	___ ___	1	2	8	1	2	1	2
05		___ ___	1	2	1	2	0 1 2 3 4 8	___ ___	1	2	8	1	2	1	2
06		___ ___	1	2	1	2	0 1 2 3 4 8	___ ___	1	2	8	1	2	1	2
07		___ ___	1	2	1	2	0 1 2 3 4 8	___ ___	1	2	8	1	2	1	2
08		___ ___	1	2	1	2	0 1 2 3 4 8	___ ___	1	2	8	1	2	1	2
09		___ ___	1	2	1	2	0 1 2 3 4 8	___ ___	1	2	8	1	2	1	2
10		___ ___	1	2	1	2	0 1 2 3 4 8	___ ___	1	2	8	1	2	1	2
11		___ ___	1	2	1	2	0 1 2 3 4 8	___ ___	1	2	8	1	2	1	2
12		___ ___	1	2	1	2	0 1 2 3 4 8	___ ___	1	2	8	1	2	1	2
13		___ ___	1	2	1	2	0 1 2 3 4 8	___ ___	1	2	8	1	2	1	2
14		___ ___	1	2	1	2	0 1 2 3 4 8	___ ___	1	2	8	1	2	1	2
15		___ ___	1	2	1	2	0 1 2 3 4 8	___ ___	1	2	8	1	2	1	2

<u>Class codes for ED5, ED10 & ED16</u>: ***Primary*** *01-05* ***Middle*** *01-03* ***Matric*** *01-02* ***Higher*** *01-07*

**EDUCATION 2**													**ED**
**ED1**.<br>*Line number*	**ED2**.<br>*Name and age.*		**ED9**.<br>At any time during the current school year (2017-18), did ***(name)*** attend school, or any PreSchool/ Katchi/ Early Childhood Education programme?<br><br>1 YES<br>2 NO ⇨ *ED15*	**ED10**.<br>During this current school year (2017-18), which level and grade or class is ***(name)*** attending?<br>LEVEL:<br>0 Preschool/ Katchi/ECE ⇨ *ED15*<br>1 PRIMARY<br>2 LOWER SEC.<br>3 UPPER SEC.<br>4 HIGHER<br>8 DK	GRADE/ CLASS:<br>98 DK	**ED10A.**<br>Is ***(name)*** currently going to any school. *(reference period is last seven days for at least 4-5 hours daily)*<br><br>1 YES<br>2 NO ⇨ *ED15*	**ED11**.<br>Is (he/she) attending a public school?<br><br>*If "Yes", record '1'. If "No", probe to code who controls and manages the school.*<br>1 GOVT./ PUBLIC<br>2 RELIGIOUS/ MISSIONARY.<br>3 PRIVATE<br>6 OTHER<br>8 DK	**ED12**.<br>In the current school year (2017-18), has ***(name)*** received any school tuition support?<br><br>*If "Yes", probe to ensure that support was not received from family, other relatives, friends or neighbours.*<br><br>1 YES<br>2 NO ⇨ *ED14*<br>8 DK ⇨ *ED14*	**ED13**.<br>Who provided the tuition support?<br><br>*Record all mentioned.*<br><br>A GOVT. / PUBLIC<br>B RELIGIOUS/ MISSIONARY.<br>C PRIVATE.<br>X OTHER<br>Z DK	**ED14**.<br>For the current school year (2017-18), has ***(name)*** received any material support or cash to buy shoes, exercise books, notebooks, school uniforms or other school supplies?<br><br>*If "Yes", probe to ensure that support was not received from family, other relatives, friends or neighbours.*<br><br>1 YES<br>2 NO<br>8 DK	**ED15**.<br>At any time during the previous school year (2016-17), did ***(name)*** attend school or any PreSchool/ Katchi/ Early Childhood Education programme?<br><br>1 YES<br>2 NO ⇨ *Next Line*<br>8 DK ⇨ *Next Line*	**ED16**.<br>During the previous school year (2016-17), which level and grade or class did ***(name)*** attend?<br>LEVEL:<br>0 Preschool/ Katchi/ ECE ⇨ *Next Line*<br>1 PRIMARY<br>2 LOWER SEC.<br>3 UPPER SEC.<br>4 HIGHER<br>8 DK	GRADE/ CLASS:<br>98 DK
LINE	NAME	AGE	YES NO	LEVEL	GRADE/ CLASS*	YES NO	AUTHORITY	YES NO DK	TUITION	YES NO DK	YES NO DK	LEVEL	GRADE/ CLASS*
01		___ ___	1 2	0 1 2 3 4 8	___ ___	1 2	1 2 3 6 8	1 2 8	A B C X Z	1 2 8	1 2 8	0 1 2 3 4 8	___ ___
02		___ ___	1 2	0 1 2 3 4 8	___ ___	1 2	1 2 3 6 8	1 2 8	A B C X Z	1 2 8	1 2 8	0 1 2 3 4 8	___ ___
03		___ ___	1 2	0 1 2 3 4 8	___ ___	1 2	1 2 3 6 8	1 2 8	A B C X Z	1 2 8	1 2 8	0 1 2 3 4 8	___ ___
04		___ ___	1 2	0 1 2 3 4 8	___ ___	1 2	1 2 3 6 8	1 2 8	A B C X Z	1 2 8	1 2 8	0 1 2 3 4 8	___ ___
05		___ ___	1 2	0 1 2 3 4 8	___ ___	1 2	1 2 3 6 8	1 2 8	A B C X Z	1 2 8	1 2 8	0 1 2 3 4 8	___ ___
06		___ ___	1 2	0 1 2 3 4 8	___ ___	1 2	1 2 3 6 8	1 2 8	A B C X Z	1 2 8	1 2 8	0 1 2 3 4 8	___ ___
07		___ ___	1 2	0 1 2 3 4 8	___ ___	1 2	1 2 3 6 8	1 2 8	A B C X Z	1 2 8	1 2 8	0 1 2 3 4 8	___ ___
08		___ ___	1 2	0 1 2 3 4 8	___ ___	1 2	1 2 3 6 8	1 2 8	A B C X Z	1 2 8	1 2 8	0 1 2 3 4 8	___ ___
09		___ ___	1 2	0 1 2 3 4 8	___ ___	1 2	1 2 3 6 8	1 2 8	A B C X Z	1 2 8	1 2 8	0 1 2 3 4 8	___ ___
10		___ ___	1 2	0 1 2 3 4 8	___ ___	1 2	1 2 3 6 8	1 2 8	A B C X Z	1 2 8	1 2 8	0 1 2 3 4 8	___ ___
11		___ ___	1 2	0 1 2 3 4 8	___ ___	1 2	1 2 3 6 8	1 2 8	A B C X Z	1 2 8	1 2 8	0 1 2 3 4 8	___ ___
12		___ ___	1 2	0 1 2 3 4 8	___ ___	1 2	1 2 3 6 8	1 2 8	A B C X Z	1 2 8	1 2 8	0 1 2 3 4 8	___ ___
13		___ ___	1 2	0 1 2 3 4 8	___ ___	1 2	1 2 3 6 8	1 2 8	A B C X Z	1 2 8	1 2 8	0 1 2 3 4 8	___ ___
14		___ ___	1 2	0 1 2 3 4 8	___ ___	1 2	1 2 3 6 8	1 2 8	A B C X Z	1 2 8	1 2 8	0 1 2 3 4 8	___ ___
15		___ ___	1 2	0 1 2 3 4 8	___ ___	1 2	1 2 3 6 8	1 2 8	A B C X Z	1 2 8	1 2 8	0 1 2 3 4 8	___ ___

Class codes for ED5, ED10 & ED16: ***Primary 01-05*** ***Middle 01-03*** ***Matric 01-02*** ***Higher 01-07***

DISABILITY 1 DA

**DA1.**<br>*Line number*	**DA2.**<br>*Name and age.*		**DA3.**<br>*Age 18 or above?*<br><br>1 YES<br>2 NO ⇨<br>*Next Line*	**DA4.**<br>Does ***(name)*** wear glasses or contact lenses to help them see?<br><br>1 YES<br>2 NO ⇨<br>*DA6*	**DA5.**<br>I would like to know if ***(name)*** has difficulty seeing even when wearing glasses or contact lenses. Would you say that ***(name)*** has no difficulty seeing, some difficulty, a lot of difficulty, or cannot see at all?<br><br>1 NO DIFFICULTY SEEING ⇨ *DA7*<br>2 SOME DIFFICULTY ⇨ *DA7*<br>3 A LOT OF DIFFICULTY ⇨ *DA7*<br>4 CANNOT SEE AT ALL ⇨ *DA7*<br>8 DON'T KNOW ⇨ *DA7*	**DA6.**<br>I would like to know if ***(name)*** has difficulty seeing. Would you say that ***(name)*** has no difficulty seeing, some difficulty, a lot of difficulty, or cannot see at all?<br><br>1 NO DIFFICULTY SEEING<br>2 SOME DIFFICULTY<br>3 A LOT OF DIFFICULTY<br>4 CANNOT SEE AT ALL<br>8 DON'T KNOW	**DA7.**<br>Does ***(name)*** wear a hearing aid?<br><br>1 YES<br>2 NO ⇨<br>*DA9*	**DA8.**<br>I would like to know if ***(name)*** has difficulty hearing even when using a hearing aid. Would you say that ***(name)*** has no difficulty hearing, some difficulty, a lot of difficulty, or cannot hear at all?<br><br>1 NO DIFFICULTY HEARING ⇨ *DA10*<br>2 SOME DIFFICULTY ⇨ *DA10*<br>3 A LOT OF DIFFICULTY ⇨ *DA10*<br>4 CANNOT HEAR AT ALL ⇨ *DA10*<br>8 DON'T KNOW ⇨ *DA10*	**DA9.**<br>I would like to know if ***(name)*** has difficulty hearing. Would you say that ***(name)*** has no difficulty hearing, some difficulty, a lot of difficulty, or cannot hear at all?<br><br>1 NO DIFFICULTY HEARING<br>2 SOME DIFFICULTY<br>3 A LOT OF DIFFICULTY<br>4 CANNOT HEAR AT ALL<br>8 DON'T KNOW	**DA10.**<br>I would like to know if ***(name)*** has difficulty communicating when using his/her usual language. Would you say that ***(name)*** has no difficulty understanding or being understood, some difficulty, a lot of difficulty, or cannot communicate at all?<br><br>1 NO DIFFICULTY COMMUNICATING<br>2 SOME DIFFICULTY<br>3 A LOT OF DIFFICULTY<br>4 CANNOT COMMUNICATE AT ALL<br>8 DON'T KNOW
LINE	NAME	AGE	Y N	Y N	SEEING	SEEING	Y N	HEARING	HEARING	COMMUNICATION
01		___ ___	1 2	1 2	1 2 3 4 8	1 2 3 4 8	1 2	1 2 3 4 8	1 2 3 4 8	1 2 3 4 8
02		___ ___	1 2	1 2	1 2 3 4 8	1 2 3 4 8	1 2	1 2 3 4 8	1 2 3 4 8	1 2 3 4 8
03		___ ___	1 2	1 2	1 2 3 4 8	1 2 3 4 8	1 2	1 2 3 4 8	1 2 3 4 8	1 2 3 4 8
04		___ ___	1 2	1 2	1 2 3 4 8	1 2 3 4 8	1 2	1 2 3 4 8	1 2 3 4 8	1 2 3 4 8
05		___ ___	1 2	1 2	1 2 3 4 8	1 2 3 4 8	1 2	1 2 3 4 8	1 2 3 4 8	1 2 3 4 8
06		___ ___	1 2	1 2	1 2 3 4 8	1 2 3 4 8	1 2	1 2 3 4 8	1 2 3 4 8	1 2 3 4 8
07		___ ___	1 2	1 2	1 2 3 4 8	1 2 3 4 8	1 2	1 2 3 4 8	1 2 3 4 8	1 2 3 4 8
08		___ ___	1 2	1 2	1 2 3 4 8	1 2 3 4 8	1 2	1 2 3 4 8	1 2 3 4 8	1 2 3 4 8
09		___ ___	1 2	1 2	1 2 3 4 8	1 2 3 4 8	1 2	1 2 3 4 8	1 2 3 4 8	1 2 3 4 8
10		___ ___	1 2	1 2	1 2 3 4 8	1 2 3 4 8	1 2	1 2 3 4 8	1 2 3 4 8	1 2 3 4 8
11		___ ___	1 2	1 2	1 2 3 4 8	1 2 3 4 8	1 2	1 2 3 4 8	1 2 3 4 8	1 2 3 4 8
12		___ ___	1 2	1 2	1 2 3 4 8	1 2 3 4 8	1 2	1 2 3 4 8	1 2 3 4 8	1 2 3 4 8
13		___ ___	1 2	1 2	1 2 3 4 8	1 2 3 4 8	1 2	1 2 3 4 8	1 2 3 4 8	1 2 3 4 8
14		___ ___	1 2	1 2	1 2 3 4 8	1 2 3 4 8	1 2	1 2 3 4 8	1 2 3 4 8	1 2 3 4 8
15		___ ___	1 2	1 2	1 2 3 4 8	1 2 3 4 8	1 2	1 2 3 4 8	1 2 3 4 8	1 2 3 4 8

DISABILITY 2							DA
**DA1.** *Line number*	**DA2.** *Name and age.*		**DA11.** I would like to know if ***(name)*** has difficulty remembering or concentrating. Would you say that ***(name)*** has no difficulty remembering or concentrating, some difficulty, a lot of difficulty, or cannot remember or concentrate at all?<br>1 NO DIFFICULTY REMEMBERING/ CONCENTRATING<br>2 SOME DIFFICULTY<br>3 A LOT OF DIFFICULTY<br>4 CANNOT REMEMBER/CONCENTRATE AT ALL<br>8 DON'T KNOW	**DA12.** I would like to know if ***(name)*** has difficulty walking or climbing steps. Would you say that ***(name)*** has no difficulty walking or climbing steps, some difficulty, a lot of difficulty, or cannot walk or climb steps at all?<br>1 NO DIFFICULTY WALKING OR CLIMBING<br>2 SOME DIFFICULTY<br>3 A LOT OF DIFFICULTY<br>4 CANNOT WALK OR CLIMB AT ALL<br>8 DON'T KNOW	**DA13.** I would like to know if ***(name)*** has difficulty washing all over or dressing. Would you say that ***(name)*** has no difficulty washing all over or dressing, some difficulty, a lot of difficulty, or cannot wash all over or dress at all?<br>1 NO DIFFICULTY WASHING OR DRESSING<br>2 SOME DIFFICULTY<br>3 A LOT OF DIFFICULTY<br>4 CANNOT WASH OR DRESS AT ALL<br>8 DON'T KNOW	**DA14.** Has ***(name)*** taken any benefit from social protection scheme due to disability/ functioning?<br>A ZAKAT & BAIT UL MAAL<br>B BISP<br>C KHIDMAT CARD<br>D PENSION / RETIRMENT<br>E WATAN / HEALTH CARD<br>F NOT ANY<br>X OTHER<br>Z DK	
LINE	NAME	AGE	MEMORY	WALKING/ CLIMBING	SELF-CARE	BENEFIT	
01		___ ___	1 2 3 4 8	1 2 3 4 8	1 2 3 4 8	A B C D E F X Z	
02		___ ___	1 2 3 4 8	1 2 3 4 8	1 2 3 4 8	A B C D E F X Z	
03		___ ___	1 2 3 4 8	1 2 3 4 8	1 2 3 4 8	A B C D E F X Z	
04		___ ___	1 2 3 4 8	1 2 3 4 8	1 2 3 4 8	A B C D E F X Z	
05		___ ___	1 2 3 4 8	1 2 3 4 8	1 2 3 4 8	A B C D E F X Z	
06		___ ___	1 2 3 4 8	1 2 3 4 8	1 2 3 4 8	A B C D E F X Z	
07		___ ___	1 2 3 4 8	1 2 3 4 8	1 2 3 4 8	A B C D E F X Z	
08		___ ___	1 2 3 4 8	1 2 3 4 8	1 2 3 4 8	A B C D E F X Z	
09		___ ___	1 2 3 4 8	1 2 3 4 8	1 2 3 4 8	A B C D E F X Z	
10		___ ___	1 2 3 4 8	1 2 3 4 8	1 2 3 4 8	A B C D E F X Z	
11		___ ___	1 2 3 4 8	1 2 3 4 8	1 2 3 4 8	A B C D E F X Z	
12		___ ___	1 2 3 4 8	1 2 3 4 8	1 2 3 4 8	A B C D E F X Z	
13		___ ___	1 2 3 4 8	1 2 3 4 8	1 2 3 4 8	A B C D E F X Z	
14		___ ___	1 2 3 4 8	1 2 3 4 8	1 2 3 4 8	A B C D E F X Z	
15		___ ___	1 2 3 4 8	1 2 3 4 8	1 2 3 4 8	A B C D E F X Z	

HOUSEHOLD CHARACTERISTICS		HC
**HC1B**. What is the mother tongue of (*name of the head of the household from HL2*)?	ENGLISH ....1<br>URDU ....2<br>PUNJABI/POTOHARI ....3<br>SARAIKI ....4<br>OTHER LANGUAGE<br>(*specify*) 6	
**HC3**. How many rooms do members of this household usually use for sleeping?	NUMBER OF ROOMS .... __ __	
**HC4**. *Main material of the dwelling floor.*<br>*Record observation.*<br>*If observation is not possible, ask the respondent to determine the material of the dwelling floor.*	**NATURAL FLOOR**<br>EARTH / SAND ....11<br>DUNG ....12<br>**FINISHED FLOOR**<br>PARQUET OR POLISHED WOOD ....31<br>VINYL OR ASPHALT STRIPS ....32<br>CERAMIC TILES/MARBLE/CHIPS ....33<br>CEMENT ....34<br>CARPET ....35<br>BRICKS FLOOR ....36<br>OTHER (*specify*) ________96	
**HC5**. *Main material of the roof.*<br>*Record observation.*	**NATURAL ROOFING**<br>NO ROOF ....11<br>THATCH / PALM LEAF ....12<br>SOD ....13<br>**RUDIMENTARY ROOFING**<br>RUSTIC MAT ....21<br>PALM / BAMBOO ....22<br>WOOD PLANKS ....23<br>**FINISHED ROOFING**<br>METAL / TIN / T-IRON / GIRDERS ....31<br>WOOD / WOODEN BEAMS ....32<br>CALAMINE / CEMENT FIBRE ....33<br>CERAMIC TILES ....34<br>CEMENT ....35<br>OTHER (*specify*) 96	
**HC6**. *Main material of the exterior walls.*<br>*Record observation.*	**NATURAL WALLS**<br>NO WALLS ....11<br>CANE / PALM / TRUNKS ....12<br>DIRT ....13<br>**RUDIMENTARY WALLS**<br>BAMBOO WITH MUD ....21<br>STONE WITH MUD ....22<br>UNCOVERED ADOBE ....23<br>PLYWOOD ....24<br>CARDBOARD ....25<br>REUSED WOOD ....26<br>**FINISHED WALLS**<br>CEMENT ....31<br>STONE WITH LIME / CEMENT ....32<br>BRICKS ....33<br>CEMENT BLOCKS ....34<br>COVERED ADOBE ....35<br>OTHER (*specify*) ________96	

**HC7.** Does your household have:<br><br>[A] A fixed telephone line?<br>[B] A radio?<br>[C] Gas Heater?<br>[D] Cooking Range?<br>[E] Sewing Machine (without electric motor)?<br>[F] An iron (Gas/ Coal)?<br>[G] Bed<br>[H] Sofa<br>[I] Cupboard<br>[J] Wall Clock	YES NO<br><br>FIXED TELEPHONE LINE..................1 2<br>RADIO..1 2<br>GAS HEATER.......................................1 2<br>COOKING RANGE ....1 2<br>SEWING MACHINE ....1 2<br>IRON ....1 2<br>BED ....1 2<br>SOFA ....1 2<br>CUPBOARD..1 2<br>WALL CLOCK 1 2	
**HC8.** Does your household have electricity?	YES, INTERCONNECTED GRID ....1<br>YES, OFF-GRID (GENERATOR/ISOLATED SYSTEM) ....2<br>NO 3	3⇨*HC10*
**HC9**. Does your household have:<br><br>[A] A television?<br>[B] A refrigerator?<br>[C] A Washing Machine/ Dryer<br>[D] An Air Cooler/ Fan<br>[E] A Microwave Oven<br>[F] An Electric Iron<br>[G] A Water Filter<br>[H] A Donkey Pump/ Turbine<br>[I] An Air conditioner<br>[J] A Sewing Machine (with electric motor)?	YES NO<br><br>TELEVISION ....1 2<br>REFRIGERATOR ....1 2<br>WASHING MACHINE/ DRYER ....1 2<br>AIR COOLER/ FAN ....1 2<br>MICROWAVE OVEN ....1 2<br>ELECTRIC IRON.................................1 2<br>WATER FILTER..................................1 2<br>DONKEY PUMP/ TURBINE ....1 2<br>AIR CONDITIONER ....1 2<br>SEWING MACHINE 1 2	
**HC10**. Does any member of your household own:<br><br>[A] A watch?<br>[B] A bicycle?<br>[C] A motorcycle or scooter?<br>[D] An animal-drawn cart?<br>[E] A car, truck, bus or van?<br>[F] A boat with a motor?<br>[G] A Tractor trolley<br>[H] An Autorickshaw/ Chingchi	YES NO<br><br>WATCH.. 1 2<br>BICYCLE .... 1 2<br>MOTORCYCLE / SCOOTER .... 1 2<br>ANIMAL-DRAWN CART .... 1 2<br>CAR / TRUCK / BUS/VAN .... 1 2<br>BOAT WITH MOTOR.......................... 1 2<br>TRACTOR TROLLEY.......................... 1 2<br>AUTORICKSHAW/ CHINGCHI 1 2	
**HC11**. Does any member of your household have a computer or a tablet?	YES...1<br>NO 2	
**HC12**. Does any member of your household have a mobile telephone?	YES...1<br>NO 2	
**HC13**. Does your household have access to internet at home?	YES...1<br>NO 2	

**HC14**. Do you or someone living in this household own this dwelling?<br><br>*If 'No', then ask:* Do you rent this dwelling from someone not living in this household?<br><br>*If 'Rented from someone else', record '2'. For other responses, record '6' and specify.*	OWN....................................1<br>RENT....................................2<br><br>OTHER (*specify*) ________________6	
**HC15**. Does any member of this household own any land that can be used for agriculture?	YES....................................1<br>NO 2	2⇨*HC17*
**HC16**. How many acres of agricultural land do members of this household own?<br><br>*If less than 1, record '00'.*<br>*1 acre = 8 kanals*	ACRES .... ___ ___<br>95 OR MORE ....95<br>DK 98	
**HC17**. Does this household own any livestock, herds, other farm animals, or poultry?	YES....................................1<br>NO 2	2⇨*HC19*
**HC18**. How many of the following animals does this household have?<br><br>[A] Milk cows, buffaloes or bulls?<br><br>[B] Other cattle?<br><br>[C] Horses, donkeys, camel or mules?<br><br>[D] Goats?<br><br>[E] Sheep?<br><br>[F] Chickens?<br><br>[H] Ducks/Turkeys?<br><br>*If none, record '00'. If 95 or more, record '95'.*<br>*If unknown, record '98'.*	MILK COWS, BUFFALOES OR BULLS…...___ ___<br><br>OTHER CATTLE……………………….…....___ ___<br><br>HORSES, DONKEYS, CAMEL OR MULES.___ ___<br><br>GOATS………………………………….…___ ___<br><br>SHEEP……………………………….….___ ___<br><br>CHICKENS…………………………..…….___ ___<br><br>DUCKS/TURKEYS…………………..………___ ___	
**HC19**. Does any member of this household have an account in a bank, post office or National Saving Centre?	YES....................................1<br>NO ....2<br>DK 8	

**SOCIAL TRANSFERS**						ST
**ST1**. I would like to ask you about various external economic assistance programmes provided to households. By external assistance I mean support that comes from the government or from non-governmental organizations such as religious, charitable, or community-based organizations. This excludes support from family, other relatives, friends or neighbours.						
	[A]<br>ZAKAT,<br>BAIT_UL_MAAL?	[B]<br>BISP?	[C]<br>KHIDMAT CARD?	[D]<br>ANY RETIREMENT / PENSION BENEFITS	[E]<br>WATAN CARD OR HEALTH CARD	[X]<br>ANY OTHER EXTERNAL ASSISTANCE PROGRAMME
**ST2.** Are you aware of (***name of programme***)?	YES.................. 1<br>NO................ 2 ⇨<br>*[B]*	YES .... 1<br>NO 2⇨<br>[C]	YES .... 1<br>NO.................... 2⇨<br>........................... [D]	YES.................. 1<br>NO.................... 2⇨<br>[E]	YES...................1<br>NO .... 2⇨<br>[X]	YES(SPECIFY)_1<br>NO.………...2⇨<br>END
**ST3**. Has your household or anyone in your household received assistance through (***name of programme***)?	YES...................1 ⇨<br>*ST4*<br>NO.....................2 ⇨<br>*[B]*<br>DK.....................8 ⇨<br>*[B]*	YES ....1 ⇨<br>*ST4*<br>NO................. 2 ⇨<br>*[C]*<br>DK..................8 ⇨<br>*[C]*	YES ....1 ⇨<br>*ST4*<br>NO..................2 ⇨<br>*[D]*<br>DK..................8 ⇨<br>*[D]*	YES................1 ⇨<br>*ST4*<br>NO..................2 ⇨<br>*[E]*<br>DK..................8 ⇨<br>*[E]*	YES................ 1 ⇨<br>*ST4*<br>NO ....2 ⇨<br>*[X]*<br>DK .... 8 ⇨<br>*[X]*	YES................1 ⇨<br>*ST4*<br>NO..................2 ⇨<br>*[End]*<br>DK..................8 ⇨<br>*[End]*
**ST4**. When was the <u>last time </u>your household or anyone in your household received assistance through (***name of programme***)?<br><br>*If less than one month, record '1' and record '00' in Months.*<br>*If less than 12 months, record '1' and record in Months.*<br>*If 1 year/12 months or more, record '2' and record in Years.*	Months Ago......**1** __ __ ⇨<br>*[B]*<br>Years Ago.....**2** __ __ ⇨<br>*[B]*<br>DK.......................998 ⇨<br>*[B]*	Months Ago......**1** __ __ ⇨<br>*[C]*<br>Years Ago..........**2** __ __ ⇨<br>*[C]*<br>DK .... 998 ⇨<br>*[C]*	Months Ago......**1** __ __ ⇨<br>*[D]*<br>Years Ago...... **2** __ __ ⇨<br>*[D]*<br>DK .... 998 ⇨<br>*[D]*	Months Ago ......**1** __ __ ⇨<br>*[E]*<br>Years Ago.....**2** __ __ ⇨<br>*[E]*<br>DK ....998 ⇨<br>*[E]*	Months Ago ......**1** __ __ ⇨<br>*[X]*<br>Years Ago .....**2** __ __ ⇨<br>*[X]*<br>DK ....998 ⇨<br>*[X]*	Months Ago......**1** __ __ ⇨<br>*[End]*<br>Years Ago.....**2** __ __ ⇨<br>*[End]*<br>DK.......................998 ⇨<br>*[End]*

REMITTANCES & CASH DONATION		RM
**RM1.** Has there been a member of this household who used to live here but is now working outside this country?	YES..1<br>NO..2	2 ⇨ RM3
**RM2**. How many members are working outside this country?	NUMBER OF PERSONS: ___ ___	
**RM3.** Did the household receive any remittance in cash from outside country/ overseas during the last year?<br><br>*Money which will not be repaid*	YES..1<br>NO..2<br><br>DK..8	
**RM4**. Did the household receive any cash donation such as zakat or other means from within the country during the last year?<br><br>*Money which will not be repaid.*<br>*Support from family, other relatives, friends or neighbours*	YES…………………………………………..1<br><br>NO……………………………………………2<br><br>DK…………………………………..……..8	

HOUSEHOLD ENERGY USE		EU
**EU1**. In your household, what type of cook stove is mainly used for cooking?	ELECTRIC STOVE 01<br>SOLAR COOKER 02<br>LIQUEFIED PETROLEUM GAS (LPG)/ COOKING GAS STOVE 03<br>PIPED NATURAL GAS STOVE 04<br>BIOGAS STOVE 05<br>LIQUID FUEL STOVE 06<br>MANUFACTURED SOLID FUEL STOVE 07<br>TRADITIONAL SOLID FUEL STOVE 08<br>THREE STONE STOVE / OPEN FIRE 09<br><br>OTHER (*specify*)__________ 96<br><br>NO FOOD COOKED IN HOUSEHOLD 97	01⇨*EU5*<br>02⇨*EU5*<br><br>03⇨*EU5*<br>04⇨*EU5*<br>05⇨*EU5*<br>06⇨*EU4*<br><br><br>09⇨*EU4*<br><br>96⇨*EU4*<br><br>97⇨*EU6*
**EU2.** Does it have a chimney?	YES 1<br>NO 2<br><br>DK 8	
**EU3.** Does it have a fan?	YES 1<br>NO 2<br><br>DK 8	
**EU4**. What type of fuel or energy source is used in this cook stove?<br><br>*If more than one, record the main energy source for this cook stove.*	ALCOHOL/ ETHANOL 01<br>GASOLINE / DIESEL 02<br>KEROSENE / PARAFFIN 03<br>COAL / LIGNITE 04<br>CHARCOAL 05<br>WOOD 06<br>CROP RESIDUE / GRASS / STRAW / SHRUBS 07<br>ANIMAL DUNG / WASTE 08<br>PROCESSED BIOMASS (PELLETS) OR WOODCHIPS 09<br>GARBAGE / PLASTIC 10<br>SAWDUST 11<br><br>OTHER (*specify*)__________ 96	
**EU5**. Is the cooking usually done in the house, in a separate building, or outdoors?<br><br>*If in main house, probe to determine if cooking is done in a separate room.*<br><br>*If outdoors, probe to determine if cooking is done on veranda, covered porch, or open air.*	IN MAIN HOUSE<br>NO SEPARATE ROOM 1<br>IN A SEPARATE ROOM 2<br><br>IN A SEPARATE BUILDING 3<br><br>OUTDOORS<br>OPEN AIR 4<br>ON VERANDA OR COVERED PORCH 5<br><br>OTHER (*specify*)__________ 6	

**EU6**. What does your household mainly use for space heating when needed?	CENTRAL HEATING .... 01<br><br>MANUFACTURED SPACE HEATER .... 02<br>TRADITIONAL SPACE HEATER .... 03<br>MANUFACTURED COOKSTOVE .... 04<br>TRADITIONAL COOKSTOVE .... 05<br>THREE STONE STOVE / OPEN FIRE .... 06<br><br>OTHER (*specify*)__________ 96<br><br>NO SPACE HEATING IN HOUSEHOLD 97	01⇨*EU8*<br><br><br><br><br><br>06⇨*EU8*<br><br>96⇨*EU8*<br><br>97⇨*EU9*
**EU7.** Does it have a chimney?	YES .... 1<br>NO .... 2<br><br>DK 8	
**EU8**. What type of fuel and energy source is used in this heater?<br><br>*If more than one, record the main energy source for this heater.*	SOLAR AIR HEATER .... 01<br>ELECTRICITY .... 02<br>PIPED NATURAL GAS .... 03<br>LIQUEFIED PETROLEUM GAS (LPG)/ COOKING GAS .... 04<br>BIOGAS .... 05<br>ALCOHOL/ ETHANOL .... 06<br>GASOLINE / DIESEL .... 07<br>KEROSENE / PARAFFIN .... 08<br>COAL / LIGNITE .... 09<br>CHARCOAL .... 10<br>WOOD .... 11<br>CROP RESIDUE / GRASS / STRAW / SHRUBS .... 12<br>ANIMAL DUNG / WASTE .... 13<br>PROCESSED BIOMASS (PELLETS) OR WOODCHIPS .... 14<br>GARBAGE / PLASTIC .... 15<br>SAWDUST .... 16<br><br>OTHER (*specify*)__________ 96	
**EU9**. At night, what does your household mainly use to light the household?	ELECTRICITY .... 01<br>SOLAR LANTERN .... 02<br>RECHARGEABLE FLASHLIGHT, TORCH OR LANTERN .... 03<br>BATTERY POWERED FLASHLIGHT, TORCH OR LANTERN .... 04<br>BIOGAS LAMP .... 05<br>GASOLINE LAMP .... 06<br>KEROSENE LAMP .... 07<br>CHARCOAL .... 08<br>WOOD .... 09<br>CROP RESIDUE / GRASS / STRAW / SHRUBS .... 10<br>ANIMAL DUNG .... 11<br>OIL LAMP .... 12<br>CANDLE .... 13<br><br>OTHER (*specify*)__________ 96<br><br>NO LIGHTING IN HOUSEHOLD 97	

WATER AND SANITATION		WS
**WS1**. What is the main source of drinking water used by members of your household?<br><br>*If unclear, probe to identify the place from which members of this household most often collect drinking water (collection point).*	**PIPED WATER**<br>PIPED INTO DWELLING .... 11<br>PIPED TO COMPOUND/ YARD / PLOT .... 12<br>PIPED TO NEIGHBOUR .... 13<br>PUBLIC TAP / STANDPIPE .... 14<br><br>**BOREHOLE**<br>TUBE WELL .... 21<br>MOTORIZED PUMP .... 22<br>HAND PUMP (MECHNICAL) .... 23<br><br>**DUG WELL**<br>PROTECTED WELL .... 31<br>UNPROTECTED WELL .... 32<br>**SPRING**<br>PROTECTED SPRING .... 41<br>UNPROTECTED SPRING .... 42<br><br>RAINWATER (POND) .... 51<br>TANKER-TRUCK .... 61<br>CART WITH SMALL TANK /DRUM/CANE ... 71<br>WATER KIOSK .... 72<br>SURFACE WATER (RIVER, DAM, LAKE, POND, STREAM, CANAL, IRRIGATION CHANNEL) .... 81<br><br>**PACKAGED WATER**<br>BOTTLED WATER .... 91<br><br>OTHER (*specify*) ________________________ 96	 11⇨*WS7*<br>12⇨*WS7*<br>13⇨*WS3*<br>14⇨*WS3*<br><br><br>21⇨*WS3*<br>22⇨*WS3*<br>23⇨*WS3*<br><br><br>31⇨*WS3*<br>32⇨*WS3*<br><br>41⇨*WS3*<br>42⇨*WS3*<br><br>51⇨*WS3*<br>61⇨*WS4*<br>71⇨*WS4*<br>72⇨*WS4*<br><br>81⇨*WS3*<br><br><br><br><br>96⇨*WS3*

**WS2**. What is the <u>main</u> source of water used by members of your household for other purposes such as cooking and handwashing?<br><br>*If unclear, probe to identify the place from which members of this household most often collect water for other purposes.*	**PIPED WATER**<br>PIPED INTO DWELLING.............................. 11<br>PIPED TO COMPOUND / YARD / PLOT...... 12<br>PIPED TO NEIGHBOUR .... 13<br>PUBLIC TAP / STANDPIPE........................... 14<br><br>**BOREHOLE**<br>TUBE WELL .... 21<br>MOTORIZED PUMP...................................... 22<br>HAND PUMP (MECHNICAL)....................... 23<br><br>**DUG WELL**<br>PROTECTED WELL .... 31<br>UNPROTECTED WELL .... 32<br>**SPRING**<br>PROTECTED SPRING.................................... 41<br>UNPROTECTED SPRING .... 42<br><br>RAINWATER... 51<br>TANKER-TRUCK .... 61<br>CART WITH SMALL TANK .... 71<br>WATER KIOSK... 72<br>SURFACE WATER (RIVER, DAM, LAKE, POND, STREAM, CANAL, IRRIGATION CHANNEL).. 81<br><br>OTHER (*specify*)________________________ 96	 11⇨*WS7*<br>12⇨*WS7*<br><br><br><br><br><br><br><br><br><br><br><br><br><br><br><br><br><br>61⇨*WS4*<br>71⇨*WS4*<br>72⇨*WS4*
**WS3**. Where is that water source located?	IN OWN DWELLING .... 1<br>IN OWN YARD / PLOT .... 2<br>ELSEWHERE 3	1⇨*WS7*<br>2⇨*WS7*
**WS4**. How long does it take for members of your household to go there, get water, and come back?	MEMBERS DO NOT COLLECT.................... 000<br><br>NUMBER OF MINUTES .... __ __ __<br><br>DK 998	000 ⇨*WS7*
**WS5**. Who usually goes to this source to collect the water for your household?<br><br>*Record the name of the person and copy the line number of this person from the LIST OF HOUSEHOLD MEMBERS Module.*	NAME___________________________________<br><br>LINE NUMBER.. __ __	
**WS6**. Since last (***day of the week***), how many times has this person collected water?	NUMBER OF TIMES................................. __ __<br><br>DK 98	
**WS7.** In the last month, has there been any time when your household did not have sufficient quantities of drinking water?	YES, AT LEAST ONCE .... 1<br>NO, ALWAYS SUFFICIENT.............................. 2<br><br>DK 8	 2⇨*WS9*<br><br>8⇨*WS9*

**WS8.** What was the main reason that you were unable to access water in sufficient quantities when needed?	WATER NOT AVAILABLE FROM SOURCE ... 1<br>WATER TOO EXPENSIVE ... 2<br>SOURCE NOT ACCESSIBLE ... 3<br><br>OTHER (*specify*) ______ 6<br><br>DK ... 8	
**WS9**. Do you or any other member of this household do anything to the water to make it safer to drink?	YES ... 1<br>NO ... 2<br><br>DK ... 8	 2⇨*WS11*<br><br>8⇨*WS11*
**WS10**. What do you usually do to make the water safer to drink?<br><br>*Probe:*<br>Anything else?<br><br>*Record all methods mentioned.*	BOIL ... A<br>ADD BLEACH / CHLORINE ... B<br>STRAIN IT THROUGH A CLOTH ... C<br>USE WATER FILTER (CERAMIC, SAND, COMPOSITE, ETC.) ... D<br>SOLAR DISINFECTION ... E<br>LET IT STAND AND SETTLE ... F<br><br>OTHER (*specify*) ______ X<br><br>DK ... Z	
**WS11**. What kind of toilet facility do members of your household usually use?<br><br>*If 'Flush' or 'Pour flush', probe:*<br>Where does it flush to?<br><br>*If not possible to determine, ask permission to observe the facility.*	**FLUSH / POUR FLUSH**<br>FLUSH TO PIPED SEWER SYSTEM ... 11<br>FLUSH TO SEPTIC TANK ... 12<br>FLUSH TO PIT LATRINE ... 13<br>FLUSH TO OPEN DRAIN ... 14<br>FLUSH TO DK WHERE ... 18<br>**PIT LATRINE**<br>VENTILATED IMPROVED PIT LATRINE ... 21<br>PIT LATRINE WITH SLAB ... 22<br>PIT LATRINE WITHOUT SLAB / OPEN PIT ... 23<br><br>BUCKET ... 41<br><br>NO FACILITY / BUSH / FIELD ... 95<br><br>OTHER (*specify*) ______ 96	 11⇨*WS14*<br><br><br>14⇨*WS14*<br>18⇨*WS14*<br><br><br><br><br><br><br>41⇨*WS14*<br><br>95⇨*End*<br><br>96⇨*WS14*
**WS12**. Has your (***answer from WS11***) ever been emptied?	YES, EMPTIED<br>WITHIN THE LAST 5 YEARS ... 1<br>MORE THAN 5 YEARS AGO ... 2<br>DON'T KNOW WHEN ... 3<br><br>NO, NEVER EMPTIED<br>NEVER REQUIRED EMPTYING ... 4<br>REPLACED WHEN FULL ... 5<br><br>DK ... 8	 <br><br><br><br><br>*4⇨WS14*<br>*5⇨WS14*<br><br>*8⇨WS14*

**WS13**. The last time it was emptied, where were the contents emptied to?<br><br>*Probe:*<br>Was it removed by a service provider?	**REMOVED BY SERVICE PROVIDER**<br>TO A TREATMENT PLANT .... 1<br>BURIED IN A COVERED PIT.......................... 2<br>TO DON'T KNOW WHERE .... 3<br><br>**EMPTIED BY HOUSEHOLD**<br>BURIED IN A COVERED PIT.......................... 4<br>TO UNCOVERED PIT, OPEN GROUND,<br>WATER BODY OR ELSEWHERE .... 5<br><br>OTHER (*specify*)__________________________ 6<br><br>DK 8	
**WS14**. Where is this toilet facility located?	IN OWN DWELLING .... 1<br>IN OWN YARD / PLOT .... 2<br>ELSEWHERE 3	
**WS15**. Do you share this facility with others who are not members of your household?	YES .... 1<br>NO 2	2⇨*End*
**WS16**. Do you share this facility only with members of other households that you know, or is the facility open to the use of the general public?	SHARED WITH KNOWN HOUSEHOLDS<br>(NOT PUBLIC) .... 1<br>SHARED WITH GENERAL PUBLIC 2	2⇨*End*
**WS17**. How many households in total use this toilet facility, including your own household?	NUMBER OF HOUSEHOLDS<br>(IF LESS THAN 10)................................... _0_ __<br><br>TEN OR MORE HOUSEHOLDS....................... 10<br><br>DK 98	

HANDWASHING		HW
**HW1**. We would like to learn about where members of this household wash their hands.<br><br>Can you please show me where members of your household most often wash their hands?<br><br>*Record result and observation.*	**OBSERVED**<br>FIXED FACILITY OBSERVED (SINK / TAP)<br>IN DWELLING ..... 1<br>IN YARD /PLOT ..... 2<br>MOBILE OBJECT OBSERVED<br>BUCKET / JUG / KETTLE ..... 3<br><br>**NOT OBSERVED**<br>NO HANDWASHING PLACE IN DWELLING / YARD / PLOT ..... 4<br>NO PERMISSION TO SEE..... 5<br><br>OTHER REASON (*specify*) ________ 6	 <br><br><br><br><br><br><br><br>4⇨*HW5*<br>5⇨*HW4*<br><br>6⇨*HW5*
**HW2**. *Observe presence of water at the place for handwashing.*<br><br>*Verify by checking the tap/pump, or basin, bucket, water container or similar objects for presence of water.*	WATER IS AVAILABLE ..... 1<br><br>WATER IS NOT AVAILABLE..... 2	
**HW3.** *Is soap or detergent present at the place for handwashing?*	YES, PRESENT..... 1<br>NO, NOT PRESENT 2	1⇨*HW7*<br>2⇨*HW5*
**HW4.** Where do you or other members of your household most often wash your hands?	FIXED FACILITY (SINK / TAP)<br>IN DWELLING ..... 1<br>IN YARD / PLOT ..... 2<br><br>MOBILE OBJECT<br>BUCKET / JUG / KETTLE ..... 3<br><br>NO HANDWASHING PLACE IN DWELLING / YARD / PLOT ..... 4<br><br>OTHER (*specify*) ________ 6	
**HW5**. Do you have any soap or detergent in your house for washing hands?	YES..... 1<br>NO..... 2	 2⇨*End*
**HW6**. Can you please show it to me?	YES, SHOWN ..... 1<br><br>NO, NOT SHOWN..... 2	 <br>2⇨*End*
**HW7**. *Record your observation.*<br><br>*Record all that apply.*	BAR OR LIQUID SOAP.....A<br>DETERGENT (POWDER / LIQUID / PASTE).......B	

SALT IODIZATION		SA
**SA1**. We would like to check whether the salt used in your household is iodized. May I have a sample of the salt used to cook meals in your household?<br><br>*Apply 2 drops of test solution, observe the darkest reaction within 30 seconds, compare to the colour chart and then record the response (1, 2 or 3) that corresponds to test outcome.*	**SALT TESTED**<br>0 PPM (NO REACTION) ....1<br>BELOW 15 PPM (BETWEEN 0 AND 15 PPM) ..2<br>ABOVE 15 PPM (AT LEAST 15 PPM)................3<br><br>**SALT NOT TESTED**<br>NO SALT IN THE HOUSE...................................4<br>OTHER REASON<br>(*specify*) ________________________ 6	 <br>2⇨*HH13*<br>3⇨*HH13*<br><br><br>4⇨*HH13*<br><br>6⇨*HH13*
**SA2**. I would like to perform one more test. May I have another sample of the same salt?<br><br>*Apply 5 drops of recheck solution. Then apply 2 drops of test solution on the same spot. Observe the darkest reaction within 30 seconds, compare to the colour chart and then record the response (1, 2 or 3) that corresponds to test outcome.*	**SALT TESTED**<br>0 PPM (NO REACTION) ....1<br>BELOW 15 PPM (BETWEEN 0 AND 15 PPM) ..2<br>ABOVE 15 PPM (AT LEAST 15 PPM)................3<br><br>**SALT NOT TESTED**<br>OTHER REASON<br>(*specify*) ________________________ 6	

**HH13**. *Record the time.*	HOUR AND MINUTES...................... __ __ : __ __	
**HH14**. *Language of the Questionnaire.*	ENGLISH ....1<br>URDU 2	
**HH15**. *Language of the Interview.*	ENGLISH ....1<br>URDU ....2<br>PUNJABI/POTOHARI...3<br>SARAIKI ....4<br><br>OTHER LANGUAGE<br>(*specify*)________________________________6	
**HH16**. *Native language of the Respondent.*	URDU ....2<br>PUNJABI/POTOHARI...3<br>SARAIKI ....4<br><br>OTHER LANGUAGE<br>(*specify*)________________________________6	
**HH17**. *Was a translator used for any parts of this questionnaire?*	YES, ENTIRE QUESTIONNAIRE..........................1<br>YES, PART OF QUESTIONNAIRE........................2<br>NO, NOT USED 3	
**HH18**. *Check HL6 in the LIST OF HOUSEHOLD MEMBERS and indicate the total number of children age 5-17 years:*	NO CHILDREN...0<br><br>1 CHILD ....1<br><br>2 OR MORE CHILDREN (NUMBER).............. __ __	0⇨*HH29*<br><br>1⇨*HH27*

HH19. *List each of the children age 5-17 years below in the order they appear in the LIST OF HOUSEHOLD MEMBERS. Do not include other household members outside of the age range 5-17 years. Record the line number, name, sex, and age for each child.*

**HH20**. *Rank number*	**HH21**. *Line number from HL1*	**HH22**. *Name from HL2*	**HH23**. *Sex from HL4*		**HH24**. *Age from HL6*
RANK	LINE	NAME	M	F	AGE
1	__ __		1	2	___ ___
2	__ __		1	2	___ ___
3	__ __		1	2	___ ___
4	__ __		1	2	___ ___
5	__ __		1	2	___ ___
6	__ __		1	2	___ ___
7	__ __		1	2	___ ___
8	__ __		1	2	___ ___

○

HH25. *Check the last digit of the household number (HH2) from the HOUSEHOLD INFORMATION PANEL. This is the number of the row you should go to in the table below.*

Check the total number of children age 5-17 years in HH18 above. This is the number of the column you should go to in the table below.

Find the box where the row and the column meet and <u>record</u> the number that appears in the box. This is the rank number (HH20) of the selected child.

LAST DIGIT OF HOUSEHOLD NUMBER (FROM HH2)	TOTAL NUMBER OF ELIGIBLE CHILDREN IN THE HOUSEHOLD (FROM HH18)						
	2	3	4	5	6	7	8+
0	2	2	4	3	6	5	4
1	1	3	1	4	1	6	5
2	2	1	2	5	2	7	6
3	1	2	3	1	3	1	7
4	2	3	4	2	4	2	8
5	1	1	1	3	5	3	1
6	2	2	2	4	6	4	2
7	1	3	3	5	1	5	3
8	2	1	4	1	2	6	4
9	1	2	1	2	3	7	5

HH26. *Record the rank number (HH20), line number (HH21), name (HH22) and age (HH24) of the selected child.*

HH27. *(When HH18=1 or when there is a single child age 5-17 in the household): Record the rank number as '1' and record the line number (HL1), the name (HL2) and age (HL6) of this child from the LIST OF HOUSEHOLD MEMBERS.*

RANK NUMBER ....__

LINE NUMBER ....__ __

NAME ____________________

AGE ....__ __

HH28. *Issue a QUESTIONNAIRE FOR CHILDREN AGE 5-17 to be administered to the mother/caretaker of this child.*

**HH29**. *Check HL8 in the LIST OF HOUSEHOLD MEMBERS: Are there any women age 15-49?*	YES, AT LEAST ONE WOMAN AGE 15-49 .... 1<br>NO 2	 2 ⇨ *HH34*
**HH30**. *Issue a separate QUESTIONNAIRE FOR INDIVIDUAL WOMEN for each woman age 15-49 years.*		
**HH31**. *Check HL6 and HL8 in the LIST OF HOUSEHOLD MEMBERS: Are there any girls age 15-17?*	YES, AT LEAST ONE GIRL AGE 15-17................. 1<br>NO 2	 2 ⇨ *HH34*
**HH32**. *Check HL20 in the LIST OF HOUSEHOLD MEMBERS: Is consent required for interviewing at least one girl age 15-17?*	YES, AT LEAST ONE GIRL AGE 15-17 WITH HL20≠90 .... 1<br>NO, HL20=90 FOR ALL GIRLS AGE 15-17 2	 <br>2 ⇨ *HH34*

HH33. As part of the survey we are also interviewing women age 15-49. We ask each person we interview for permission. A female interviewer conducts these interviews.

For girls age 15-17 we must also get permission from an adult to interview them. As mentioned before, all the information we obtain will remain strictly confidential and anonymous.

May we interview (***name(s) of female member(s) age 15-17***) later?

☐ *'Yes' for all girls age 15-17 ⇨ Continue with HH34.*

☐ *'No' for at least one girl age 15-17 and 'Yes' to at least one girl age 15-17 ⇨ Record '06' in WM17 (also in UF17 and FS17, if applicable) on individual questionnaires for those adult consent was not given. Then continue with HH34.*

☐ *'No' for all girls age 15-17 ⇨ Record '06' in WM17 (also in UF17 and FS17, if applicable) on all individual questionnaires for whom adult consent was not given. Then continue with HH34.*

**HH34**. *Check HH8 in the HOUSEHOLD INFORMATION PANEL: Is the household selected for Questionnaire for Men?*	YES, HH8=1 .... 1<br>NO, HH8=0 2	 2 ⇨ *HH40*
**HH35**. *Check HL9 in the LIST OF HOUSEHOLD MEMBERS: Are there any men age 15-49?*	YES, AT LEAST ONE MAN AGE 15-49................. 1<br>NO 2	 2 ⇨ *HH40*
**HH36**. *Issue a separate QUESTIONNAIRE FOR INDIVIDUAL MEN for each man age 15-49 years.*		
**HH37**. *Check HL6 and HL8 in the LIST OF HOUSEHOLD MEMBERS: Are there any boys age 15-17?*	YES, AT LEAST ONE BOY AGE 15-17 .... 1<br>NO 2	 2 ⇨ *HH40*
**HH38**. *Check HL20 in the LIST OF HOUSEHOLD MEMBERS: Is consent required for interviewing at least one boy age 15-17?*	YES, AT LEAST ONE BOY AGE 15-17 WITH HL20≠90 .... 1<br>NO, HL20=90 FOR ALL BOYS AGE 15-17............ 2	 <br>2 ⇨ *HH40*

HH39. As part of the survey we are also interviewing men age 15-49. We ask each person we interview for permission. A male interviewer conducts these interviews.

For boys age 15-17 we must also get permission from an adult to interview them. As mentioned before, all the information we obtain will remain strictly confidential and anonymous.

May we interview (***name(s) of male member(s) age 15-17***) later?

☐ *'Yes' for all boys age 15-17 ⇨ Continue with HH40.*

☐ *'No' for at least one boy age 15-17 and 'Yes' to at least one boy age 15-17 ⇨ Record '06' in MWM17 (also in UF17 and FS17, if applicable) on individual questionnaires for those adult consent was not given. Then continue with HH40.*

☐ *'No' for all boys age 15-17 ⇨ Record '06' in MWM17 (also in UF17 and FS17, if applicable) on all individual questionnaires for whom adult consent was not given. Then continue with HH40.*

**HH40**. *Check HL10 in the LIST OF HOUSEHOLD MEMBERS: Are there any children age 0-4?*	YES, AT LEAST ONE .... 1<br>NO.. 2	 2⇨*HH42*
**HH41**. *Issue a separate QUESTIONNAIRE FOR CHILDREN UNDER FIVE for each child age 0-4 years.*		
**HH42**. *Check HH9 in the HOUSEHOLD INFORMATION PANEL: Is the household selected for Water Quality Testing Questionnaire?*	YES, HH9=1 .... 1<br>NO, HH9=2... 2	 2⇨*HH45*
**HH43**. *Issue a separate WATER QUALITY TESTING QUESTIONNAIRE for this household*		
**HH44**. As part of the survey we are also looking at the quality of drinking water. We would like to do a simple test of your drinking water. A colleague will come and collect the water samples. May we do such a test?<br><br>*If the respondent requests to learn the results, explain that results will not be shared with individual households but will be made available to local authorities.*	YES, PERMISSION IS GIVEN........................... 1<br>NO, PERMISSION IS NOT GIVEN 2	 2⇨*Record '02' in WQ31 on the WATER QUALITY TESTING QUESTION-NAIRE*

HH45. *Now return to the HOUSEHOLD INFORMATION PANEL and,*

- *Record '01' in question HH46 (Result of the Household Questionnaire interview),*
- *Record the name and the line number (from the LIST OF HOUSEHOLD MEMBERS) of the Respondent to the Household Questionnaire interview in HH47,*
- *Fill the questions HH48 – HH52,*
- *Thank the respondent for his/her cooperation and then*
- *Proceed with the administration of the remaining individual questionnaire(s) in this household.*

If there is no individual questionnaire and no WATER QUALITY TESTING QUESTIONNAIRE to be completed in this household thank the respondent for his/her cooperation and move to the next household you have been assigned by your supervisor.

INTERVIEWER'S OBSERVATIONS

SUPERVISOR'S OBSERVATIONS
